# Supplementary material for: Body size and composition and risk of site-specific cancers in the UK Biobank and large international consortia: A mendelian randomisation study
Source: PLoS Med. 2021 Jul 29;18(7):e1003706. doi: 10.1371/journal.pmed.1003706 (PMC8320991; doi:10.1371/journal.pmed.1003706)
Supplement: S1 Checklist — STROBE, Strengthening the Reporting of Observational Studies in Epidemiology. (DOCX) [file pmed.1003706.s001.docx]

STROBE Statement—Checklist of items that should be included in reports of ***case-control studies***

|  | Item No | Recommendation | Page No |
| --- | --- | --- | --- |
| **Title and abstract** | 1 | (*a*) Indicate the study’s design with a commonly used term in the title or the abstract | Title |
|  |  | (*b*) Provide in the abstract an informative and balanced summary of what was done and what was found | Abstract |
| Introduction | | | |
| Background/rationale | 2 | Explain the scientific background and rationale for the investigation being reported | Background, para 4 |
| Objectives | 3 | State specific objectives, including any prespecified hypotheses | Background, para 4 |
| Methods | | | |
| Study design | 4 | Present key elements of study design early in the paper | Methods, Study population para 1 |
| Setting | 5 | Describe the setting, locations, and relevant dates, including periods of recruitment, exposure, follow-up, and data collection | Methods, Study population para 1 |
| Participants | 6 | (*a*) Give the eligibility criteria, and the sources and methods of case ascertainment and control selection. Give the rationale for the choice of cases and controls | Methods, Study population para 1 plus Supp Figure S1 |
|  |  | (*b*) For matched studies, give matching criteria and the number of controls per case | N/A |
| Variables | 7 | Clearly define all outcomes, exposures, predictors, potential confounders, and effect modifiers. Give diagnostic criteria, if applicable | Methods, Study population para 1 plus Supp Table S1 |
| Data sources/ measurement | 8* | For each variable of interest, give sources of data and details of methods of assessment (measurement). Describe comparability of assessment methods if there is more than one group | Methods, Study population para 1 |
| Bias | 9 | Describe any efforts to address potential sources of bias | Methods, Genetic instruments |
| Study size | 10 | Explain how the study size was arrived at | Methods, Study population para 1 |
| Quantitative variables | 11 | Explain how quantitative variables were handled in the analyses. If applicable, describe which groupings were chosen and why | Methods, Statistical analysis para 1 |
| Statistical methods | 12 | (*a*) Describe all statistical methods, including those used to control for confounding | Methods, Statistical analysis |
|  |  | (*b*) Describe any methods used to examine subgroups and interactions | Methods, Statistical analysis |
|  |  | (*c*) Explain how missing data were addressed | Methods, Study population para 1 |
|  |  | (*d*) If applicable, explain how matching of cases and controls was addressed | NA |
|  |  | (*e*) Describe any sensitivity analyses | Methods, Statistical analysis, para 1 |
| Results | | | |
| Participants | 13* | (a) Report numbers of individuals at each stage of study—eg numbers potentially eligible, examined for eligibility, confirmed eligible, included in the study, completing follow-up, and analysed | Results, para 1 plus Supp Figure S1 |
|  |  | (b) Give reasons for non-participation at each stage | Supp Figure S1 |
|  |  | (c) Consider use of a flow diagram | Sup Figure S1 |
| Descriptive data | 14* | (a) Give characteristics of study participants (eg demographic, clinical, social) and information on exposures and potential confounders | Results, para 1 plus Table 1 |
|  |  | (b) Indicate number of participants with missing data for each variable of interest | Table 1 |
| Outcome data | 15* | Report numbers in each exposure category, or summary measures of exposure | Results (all paras) |

| Main results | | 16 | (*a*) Give unadjusted estimates and, if applicable, confounder-adjusted estimates and their precision (eg, 95% confidence interval). Make clear which confounders were adjusted for and why they were included | Results (all paras) |
| --- | --- | --- | --- | --- |
|  |  |  | (*b*) Report category boundaries when continuous variables were categorized | NA |
|  |  |  | (*c*) If relevant, consider translating estimates of relative risk into absolute risk for a meaningful time period | NA |
| Other analyses | 17 | Report other analyses done—eg analyses of subgroups and interactions, and sensitivity analyses | | Results (all paras) plus Supp Figure S3 and Supp Tables S5 and S6 |
| Discussion | | | | |
| Key results | 18 | Summarise key results with reference to study objectives | | Discussion para 1 |
| Limitations | 19 | Discuss limitations of the study, taking into account sources of potential bias or imprecision. Discuss both direction and magnitude of any potential bias | | Discussion para 8 |
| Interpretation | 20 | Give a cautious overall interpretation of results considering objectives, limitations, multiplicity of analyses, results from similar studies, and other relevant evidence | | Discussion para 9 |
| Generalisability | 21 | Discuss the generalisability (external validity) of the study results | | Discussion para 8 |
| Other information | | | | |
| Funding | 22 | Give the source of funding and the role of the funders for the present study and, if applicable, for the original study on which the present article is based | | 18 |

*Give information separately for cases and controls.

**Note:** An Explanation and Elaboration article discusses each checklist item and gives methodological background and published examples of transparent reporting. The STROBE checklist is best used in conjunction with this article (freely available on the Web sites of PLoS Medicine at http://www.plosmedicine.org/, Annals of Internal Medicine at http://www.annals.org/, and Epidemiology at http://www.epidem.com/). Information on the STROBE Initiative is available at http://www.strobe-statement.org.
