# Supplementary figures and images for: Body size and composition and risk of site-specific cancers in the UK Biobank and large international consortia: A mendelian randomisation study
Source: PLoS Med. 2021 Jul 29;18(7):e1003706. doi: 10.1371/journal.pmed.1003706 (PMC8320991; doi:10.1371/journal.pmed.1003706)

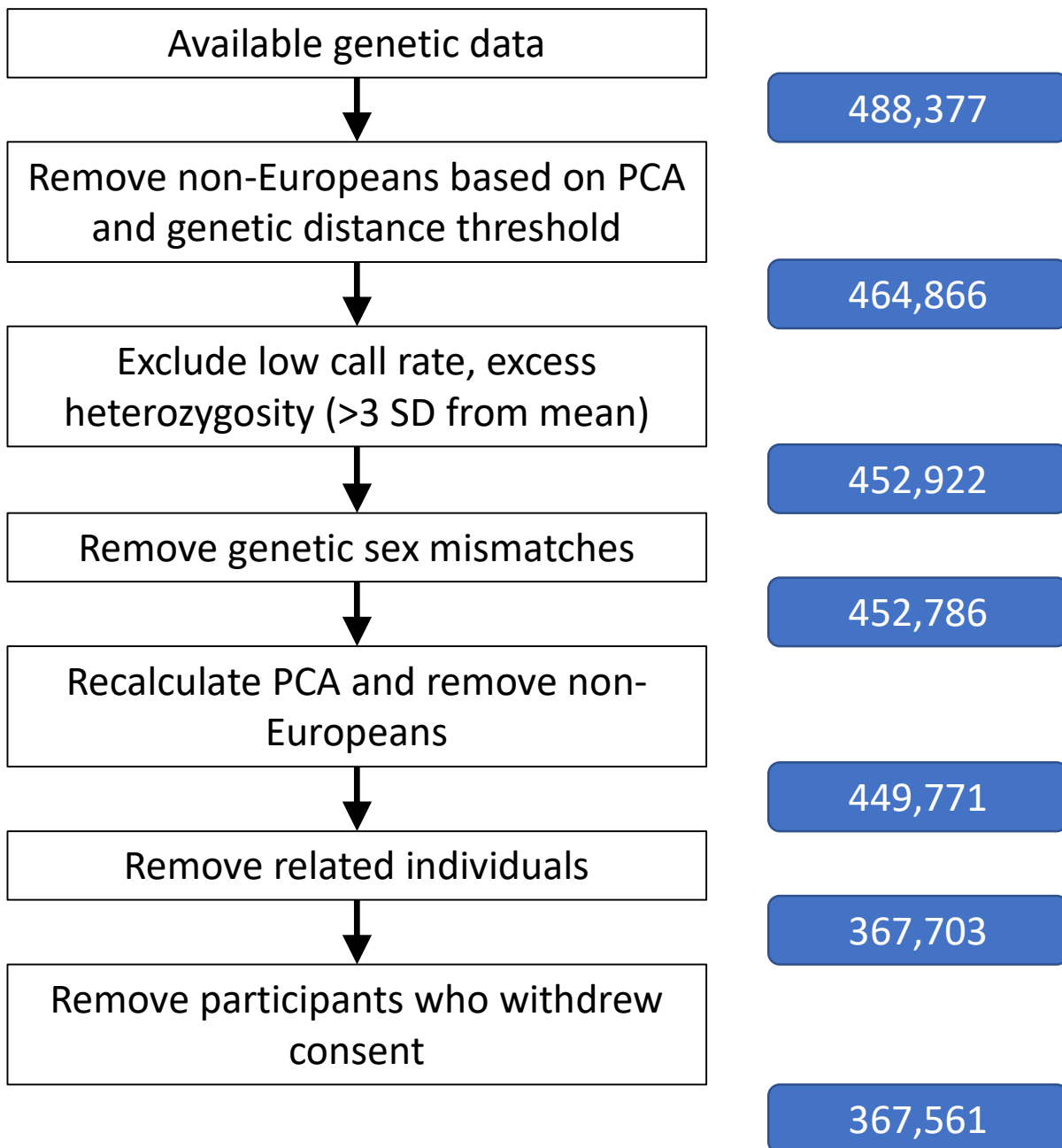

Supplement: S1 Fig — PCA, principal component analysis; SD, standard deviation; UKBB, UK Biobank. (PDF) [file pmed.1003706.s002.pdf]

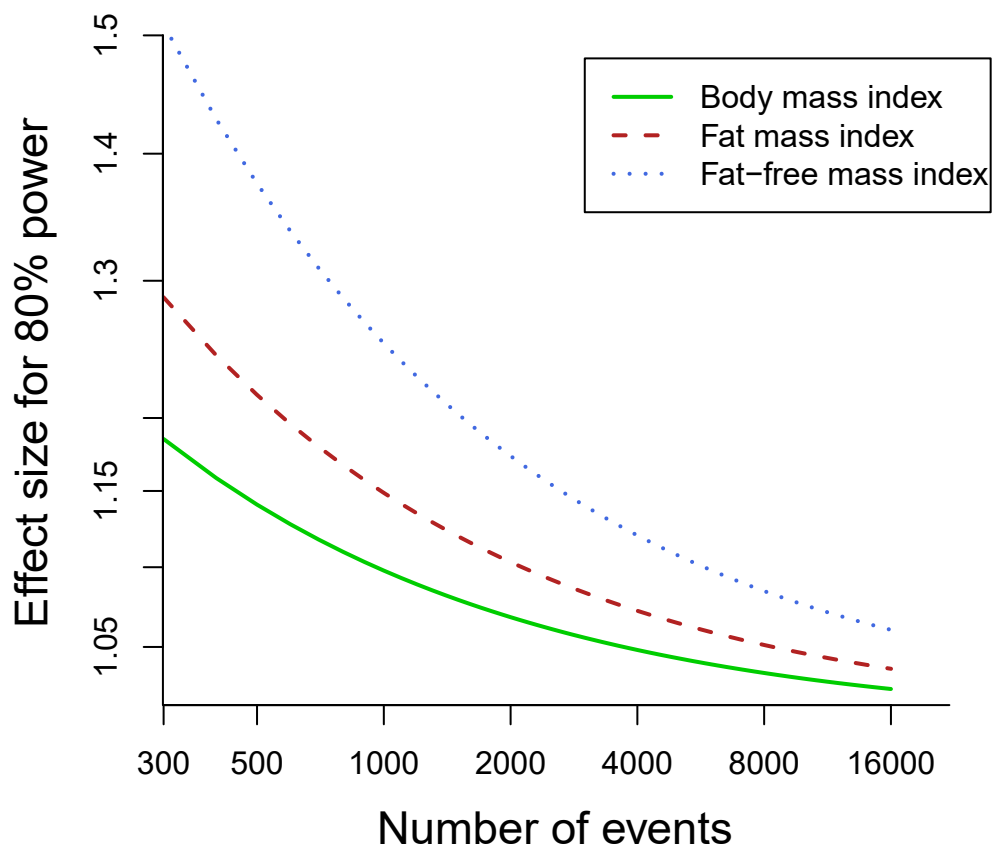

Supplement: S2 Fig — As power calculators are not available for multivariable MR, all calculations are performed for univariable MR analyses based on each risk factor in turn. BMI, body mass index; FFMI, fat-free mass index; FMI, fat mass index; MR, mendelian randomisation; OR, odds ratio. (PDF) [file pmed.1003706.s003.pdf]

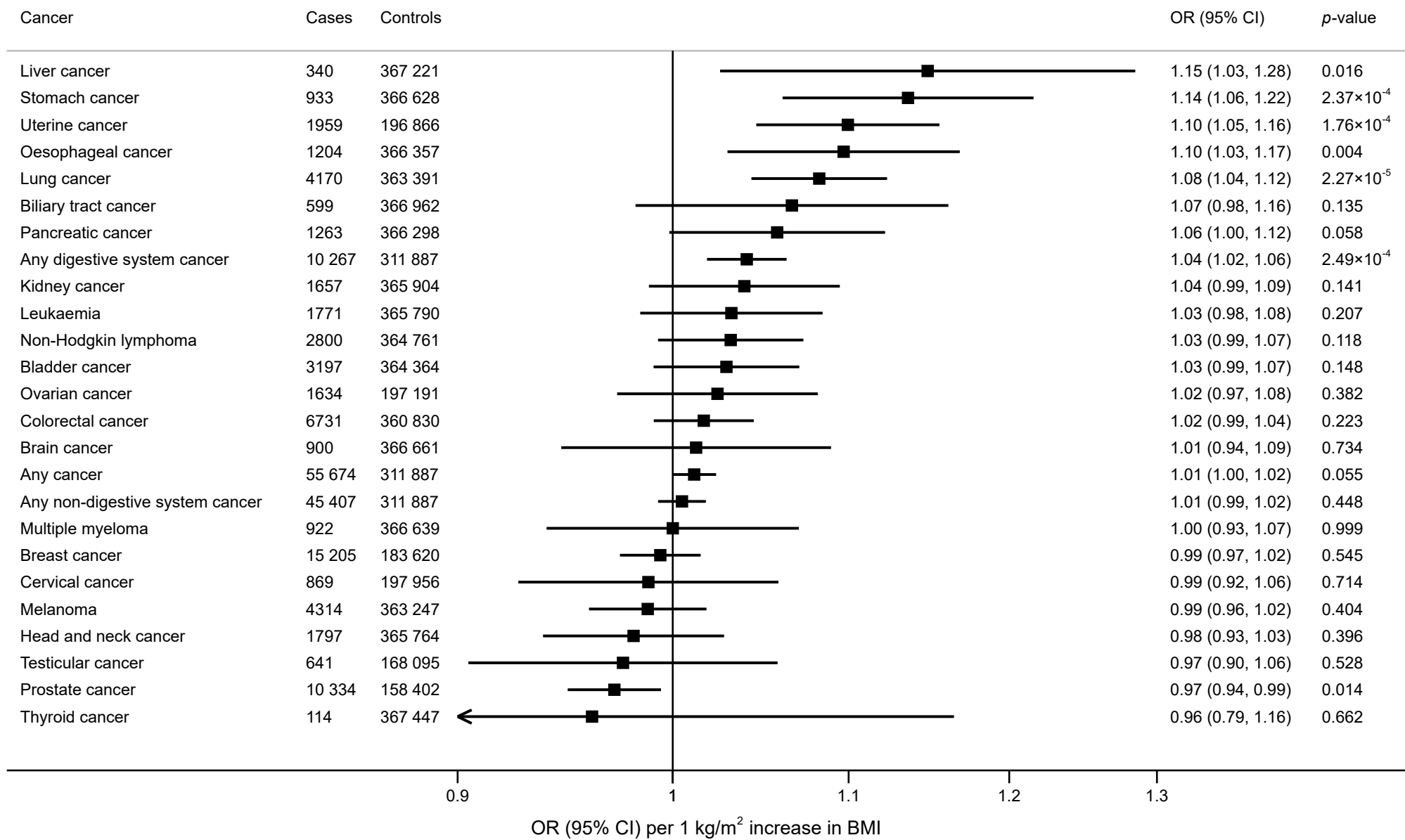

Supplement: S3 Fig — ORs are expressed per 1 kg/m2 increase in BMI. Results are obtained from the random-effects inverse-variance weighted method. BMI, body mass index; CI, confidence interval; OR, odds ratio; UKBB, UK Biobank. (PDF) [file pmed.1003706.s004.pdf]
