# Supplementary material for: Body size and composition and risk of site-specific cancers in the UK Biobank and large international consortia: A mendelian randomisation study
Source: PLoS Med. 2021 Jul 29;18(7):e1003706. doi: 10.1371/journal.pmed.1003706 (PMC8320991; doi:10.1371/journal.pmed.1003706)
Supplement: S1 Table — (PDF) [file pmed.1003706.s005.pdf]

Table S1. Sources and definition of cancers in UK Biobank

Cancer	ICD-9 codes	ICD-10 codes	Self reported cancers to nurses	Cancer Histology
Breast cancer	174, 175, V10.3	C50, Z85.3	1002	
Prostate cancer	185, V10.46	C61, Z85.46	1044	
Lung cancer	162, V10.1	C33, C34, C39.9, Z85.1	1001, 1027, 1028, 1080	
Colorectal cancer	153, 154.0, 154.1, V10.05, V10.06	C18, C19, C20, Z85.038, Z85.048	1020, 1022, 1023	
Melanoma	172, V10.82	C43, Z85.820	1059	
Non-Hodgkin lymphoma	200, 202.0 , 202.1, 202.2, 202.7, V10.71	C82, C83, C84, C85, C86, C88.0, C88.4, Z85.72	1053	
Kidney cancer	189.0, V10.52	C64, Z85.528	1034	
Head and neck cancer	140, 141, 142, 143, 144, 145,146, 147, 148, 149, 160, 161, V10.01, V10.02, V10.21, V10.22,	C00, C01, C02, C03, C04, C05, C06, C07, C08, C09, C10, C11, C12, C13, C14, C30, C31, C32, Z85.21, Z85.22, Z85.81	1006, 1007, 1009, 1004, 1010, 1011, 1012, 1077, 1078, 1079, 1005, 1015, 1016,	
Brain cancer	191, 192.0, 192.1, 192.2, 192.3, V10.85	C70, C71, C72.0, C72.3, Z85.841	1031, 1032, 1033	
Bladder cancer	188, 189.1, 189.2, V10.51, V10.53	C67, C65, C66, Z85.51, Z85.54, Z85.53	1035	
Pancreatic cancer	157	C25, Z85.07	1034	
Uterine cancer	179, 182, V10.42,	C54, C55, Z85.42	1040	
Leukaemia	204, 205, 206, 207, 208, V10.6	C91, C92, C93, C94.0, C94.2, C94.3, C94.4, C94.8, C95, Z85.6	1048, 1055, 1056, 1074	
Oesophageal cancer	150, V10.03	C15, Z85.01	1017	
Ovarian cancer	183.0, 183.2, 183.8, 183.9, V10.43	C56, C57.0, C57.4, Z85.43	1039	
Stomach cancer	151, V10.04	C16, Z85.028	1018	
Liver cancer	155.0	C22.0	1024	8170, 8171, 8172, 8173, 8174, 8175
Myeloma	203.0, 203.1,	C90.0, C90.1	1050	9732, 9733
Thyroid cancer	193, V10.87	C73. Z85.850	1065	
Biliary tract cancer	155.1, 156.0	C22.1, C23, C24	1025	
Cervical cancer	180, V10.41	C53, Z85.41	1041	
Testicular cancer	186, V10.47	C62, Z85.47	1045	
