# Supplementary material for: Body size and composition and risk of site-specific cancers in the UK Biobank and large international consortia: A mendelian randomisation study
Source: PLoS Med. 2021 Jul 29;18(7):e1003706. doi: 10.1371/journal.pmed.1003706 (PMC8320991; doi:10.1371/journal.pmed.1003706)
Supplement: S2 Table — (PDF) [file pmed.1003706.s006.pdf]

Table S2. Single nucleotide polymorphisms used in the analyses of body mass index

Exposure	SNP	Effect allele	Other allele	Beta	SE	*P* value
BMI	rs10044136	G	C	0.016	0.002	3.060e-21
BMI	rs10099330	G	A	0.012	0.002	3.220e-12
BMI	rs10116186	G	A	0.012	0.002	1.620e-09
BMI	rs10132280	C	A	0.021	0.002	2.280e-33
BMI	rs10203386	A	T	0.032	0.002	1.430e-78
BMI	rs1030015	T	G	0.011	0.002	1.160e-10
BMI	rs1045411	C	T	0.014	0.002	6.660e-14
BMI	rs1048303	C	T	0.011	0.002	1.490e-10
BMI	rs10497870	A	G	0.012	0.002	1.970e-13
BMI	rs10499694	A	G	0.013	0.002	1.290e-15
BMI	rs10510419	G	T	0.017	0.002	2.230e-13
BMI	rs10518269	C	T	0.017	0.002	4.690e-14
BMI	rs10744146	G	A	0.011	0.002	2.540e-11
BMI	rs10761247	G	A	0.011	0.002	4.530e-11
BMI	rs10761785	G	T	0.013	0.002	3.470e-16
BMI	rs10823893	A	G	0.012	0.002	1.550e-10
BMI	rs10858334	G	C	0.015	0.003	4.910e-09
BMI	rs10887584	A	G	0.013	0.002	4.570e-11
BMI	rs10920678	A	G	0.015	0.002	7.150e-20
BMI	rs10929925	C	A	0.014	0.002	3.050e-18
BMI	rs10938397	G	A	0.032	0.002	2.420e-86
BMI	rs10942267	A	G	0.015	0.002	6.530e-16
BMI	rs10954772	T	C	0.016	0.002	5.330e-14
BMI	rs10984756	G	C	0.018	0.003	4.370e-10
BMI	rs11047132	G	T	0.023	0.003	1.590e-13
BMI	rs1106761	A	G	0.013	0.002	1.750e-11
BMI	rs1106908	G	A	0.016	0.002	7.600e-23
BMI	rs11075489	C	T	0.012	0.002	1.230e-11
BMI	rs11105839	T	A	0.011	0.002	1.250e-11
BMI	rs11115176	T	C	0.013	0.002	6.790e-12
BMI	rs11121210	C	T	0.011	0.002	2.370e-10
BMI	rs11170468	A	C	0.013	0.002	1.120e-11
BMI	rs11218510	G	A	0.014	0.002	6.790e-13
BMI	rs112566467	T	C	0.018	0.002	5.860e-15
BMI	rs11525873	T	C	0.023	0.003	2.980e-13
BMI	rs11577094	T	C	0.019	0.003	3.280e-10
BMI	rs11611246	T	G	0.022	0.002	2.040e-28
BMI	rs11633626	C	A	0.016	0.002	7.270e-19
BMI	rs11636611	T	C	0.010	0.002	8.850e-10
BMI	rs11655587	C	T	0.021	0.002	6.870e-26
BMI	rs11672660	C	T	0.034	0.002	6.830e-60
BMI	rs1167821	T	A	0.020	0.002	9.380e-26

BMI	rs11692326	T	C	0.015	0.002	1.690e-14
BMI	rs11739877	T	C	0.012	0.002	4.010e-11
BMI	rs11757278	T	C	0.013	0.002	6.920e-13
BMI	rs11836108	A	G	0.011	0.002	8.370e-10
BMI	rs11856579	G	A	0.016	0.002	1.380e-16
BMI	rs11866815	C	T	0.015	0.002	2.210e-16
BMI	rs11915371	C	A	0.015	0.002	2.290e-13
BMI	rs12049202	T	C	0.024	0.002	3.350e-29
BMI	rs12072739	G	A	0.017	0.002	1.500e-13
BMI	rs12098284	T	C	0.018	0.003	9.870e-13
BMI	rs12121950	T	G	0.019	0.002	7.090e-24
BMI	rs12140153	G	T	0.035	0.003	1.440e-25
BMI	rs12151152	G	A	0.021	0.002	6.510e-27
BMI	rs12206094	C	T	0.014	0.002	1.930e-15
BMI	rs12282785	C	A	0.016	0.002	1.430e-11
BMI	rs12286929	G	A	0.018	0.002	1.930e-27
BMI	rs12321904	T	G	0.010	0.002	2.690e-09
BMI	rs12364470	G	T	0.019	0.002	2.180e-17
BMI	rs12369179	C	T	0.034	0.003	2.320e-28
BMI	rs12422552	G	C	0.013	0.002	1.780e-11
BMI	rs12429545	A	G	0.031	0.002	1.420e-37
BMI	rs12438629	C	G	0.035	0.005	2.090e-11
BMI	rs12449219	G	C	0.016	0.003	1.440e-10
BMI	rs12462975	A	G	0.019	0.002	1.470e-25
BMI	rs12509234	C	T	0.012	0.002	3.890e-10
BMI	rs12628051	T	C	0.016	0.002	2.900e-19
BMI	rs12628891	C	T	0.012	0.002	5.850e-10
BMI	rs12635553	A	T	0.010	0.002	4.100e-09
BMI	rs12680842	A	G	0.014	0.002	3.410e-16
BMI	rs12681792	A	C	0.015	0.002	2.880e-12
BMI	rs1269175	A	G	0.011	0.002	8.090e-10
BMI	rs12692596	T	C	0.012	0.002	1.030e-12
BMI	rs12714199	C	T	0.014	0.002	3.220e-16
BMI	rs12765914	T	C	0.023	0.003	1.960e-13
BMI	rs1277733	T	C	0.012	0.002	4.300e-09
BMI	rs12868881	A	T	0.014	0.002	1.400e-15
BMI	rs12922346	C	G	0.013	0.002	1.480e-11
BMI	rs12939549	A	G	0.018	0.002	3.680e-28
BMI	rs13002946	T	A	0.018	0.002	3.900e-20
BMI	rs13021737	G	A	0.058	0.002	2.89e-161
BMI	rs13033310	A	G	0.015	0.002	3.400e-11
BMI	rs13107325	T	C	0.047	0.003	3.810e-47
BMI	rs13110266	G	A	0.012	0.002	3.960e-14
BMI	rs13174863	G	A	0.020	0.002	1.940e-17
BMI	rs13191362	A	G	0.024	0.003	4.080e-21
BMI	rs1320251	C	T	0.018	0.002	5.510e-25

BMI	rs1346841	G	A	0.013	0.002	3.180e-13
BMI	rs1356506	T	C	0.014	0.002	8.390e-15
BMI	rs1383592	A	G	0.012	0.002	4.920e-09
BMI	rs140733155	G	A	0.056	0.009	2.970e-09
BMI	rs1409818	T	C	0.020	0.003	2.590e-12
BMI	rs1431659	A	G	0.019	0.002	2.330e-23
BMI	rs1436344	C	G	0.015	0.002	1.070e-17
BMI	rs1437842	G	A	0.011	0.002	8.490e-10
BMI	rs1451077	G	A	0.017	0.002	1.430e-18
BMI	rs1451109	G	A	0.016	0.002	1.360e-19
BMI	rs1470545	T	C	0.037	0.004	5.600e-18
BMI	rs1471212	A	T	0.011	0.002	1.510e-10
BMI	rs1481012	A	G	0.019	0.003	5.110e-13
BMI	rs1491905	T	C	0.015	0.002	3.920e-18
BMI	rs1501673	A	G	0.029	0.003	2.730e-31
BMI	rs1522569	T	G	0.014	0.002	1.600e-10
BMI	rs1536053	C	T	0.012	0.002	8.020e-11
BMI	rs1561554	G	C	0.012	0.002	4.890e-11
BMI	rs1658820	T	G	0.013	0.002	3.770e-11
BMI	rs16851483	T	G	0.035	0.003	4.870e-25
BMI	rs16989232	A	G	0.012	0.002	6.190e-12
BMI	rs17001561	A	G	0.015	0.002	1.810e-10
BMI	rs17024393	C	T	0.064	0.005	7.110e-39
BMI	rs17201143	C	T	0.011	0.002	1.100e-09
BMI	rs17276464	T	C	0.010	0.002	4.810e-09
BMI	rs1730859	G	A	0.012	0.002	2.960e-12
BMI	rs17405819	T	C	0.021	0.002	6.040e-33
BMI	rs17636031	C	T	0.015	0.002	3.870e-17
BMI	rs17681451	G	A	0.023	0.003	7.370e-13
BMI	rs17806224	G	A	0.026	0.002	7.910e-32
BMI	rs1852006	G	A	0.015	0.002	6.710e-18
BMI	rs1884389	C	T	0.011	0.002	3.720e-10
BMI	rs1884429	T	C	0.013	0.002	2.300e-12
BMI	rs1884897	G	A	0.018	0.002	2.700e-28
BMI	rs1899689	T	C	0.012	0.002	4.200e-13
BMI	rs1916801	A	T	0.017	0.002	1.930e-24
BMI	rs1927790	C	T	0.014	0.002	1.570e-17
BMI	rs1928295	T	C	0.013	0.002	2.230e-16
BMI	rs194809	A	G	0.013	0.002	4.860e-09
BMI	rs1951455	C	T	0.015	0.002	6.050e-15
BMI	rs1954494	T	C	0.010	0.002	2.430e-09
BMI	rs1982350	G	A	0.016	0.002	1.980e-18
BMI	rs1982441	T	G	0.017	0.003	2.170e-11
BMI	rs1996120	G	A	0.017	0.002	3.880e-24
BMI	rs2033529	G	A	0.020	0.002	2.050e-29
BMI	rs2051559	C	T	0.017	0.003	3.780e-11

BMI	rs2053682	A	C	0.017	0.002	2.590e-20
BMI	rs2065418	T	G	0.014	0.002	6.070e-15
BMI	rs2112347	T	G	0.028	0.002	1.170e-61
BMI	rs2134858	C	T	0.012	0.002	5.870e-12
BMI	rs215614	G	A	0.014	0.002	2.770e-16
BMI	rs217669	C	T	0.017	0.002	6.270e-16
BMI	rs2183824	T	C	0.023	0.002	2.290e-41
BMI	rs2192158	A	G	0.014	0.002	7.420e-16
BMI	rs2196618	G	A	0.014	0.002	1.490e-12
BMI	rs2206277	T	C	0.041	0.002	1.820e-83
BMI	rs2228213	G	A	0.014	0.002	5.500e-17
BMI	rs2246012	C	T	0.016	0.002	1.150e-13
BMI	rs2342892	T	G	0.013	0.002	1.330e-13
BMI	rs2357760	A	G	0.014	0.002	2.110e-16
BMI	rs2391540	T	A	0.014	0.002	1.360e-14
BMI	rs2396625	T	A	0.018	0.002	2.810e-24
BMI	rs2400414	C	T	0.013	0.002	5.520e-13
BMI	rs2439823	G	A	0.017	0.002	6.510e-22
BMI	rs2601777	G	A	0.014	0.002	2.840e-17
BMI	rs2605603	G	A	0.010	0.002	2.040e-10
BMI	rs2619976	T	C	0.011	0.002	1.990e-09
BMI	rs2622274	G	T	0.011	0.002	3.230e-10
BMI	rs264962	C	G	0.012	0.002	1.740e-12
BMI	rs2715423	G	A	0.012	0.002	1.900e-09
BMI	rs273505	C	T	0.017	0.002	3.320e-19
BMI	rs2777768	A	G	0.012	0.002	6.380e-10
BMI	rs2820295	A	G	0.024	0.002	5.560e-39
BMI	rs2832283	A	G	0.012	0.002	4.720e-09
BMI	rs28350	A	G	0.017	0.002	1.070e-14
BMI	rs2861089	A	T	0.011	0.002	1.550e-09
BMI	rs2861685	T	C	0.017	0.002	7.820e-18
BMI	rs2862996	G	T	0.022	0.002	3.600e-35
BMI	rs288230	T	C	0.024	0.002	4.670e-26
BMI	rs2907948	G	A	0.015	0.002	1.950e-14
BMI	rs2968487	T	C	0.018	0.002	7.550e-22
BMI	rs3101336	C	T	0.025	0.002	4.800e-54
BMI	rs34277166	A	G	0.014	0.002	8.260e-14
BMI	rs34811474	G	A	0.029	0.002	8.500e-38
BMI	rs349088	C	A	0.013	0.002	3.500e-14
BMI	rs354508	C	T	0.015	0.002	2.690e-10
BMI	rs355777	C	G	0.015	0.002	2.130e-18
BMI	rs35851183	G	A	0.012	0.002	1.070e-09
BMI	rs36061954	T	C	0.012	0.002	1.050e-09
BMI	rs3739514	A	G	0.013	0.002	6.640e-11
BMI	rs3744017	A	G	0.014	0.002	2.430e-10
BMI	rs3770799	G	A	0.011	0.002	1.210e-10

BMI	rs3806114	G	A	0.012	0.002	1.460e-11
BMI	rs3808477	C	T	0.018	0.002	8.730e-22
BMI	rs3811125	C	T	0.015	0.002	9.420e-13
BMI	rs3825061	T	C	0.014	0.002	6.150e-16
BMI	rs3923783	C	A	0.022	0.002	4.120e-23
BMI	rs3930349	C	A	0.014	0.002	7.680e-12
BMI	rs39654	G	A	0.016	0.002	1.750e-21
BMI	rs4097319	T	G	0.011	0.002	4.320e-10
BMI	rs4123853	T	C	0.014	0.002	1.060e-15
BMI	rs41284828	G	A	0.034	0.006	3.000e-09
BMI	rs4256980	G	C	0.019	0.002	8.630e-29
BMI	rs427943	C	A	0.018	0.002	3.600e-25
BMI	rs4290163	T	G	0.013	0.002	3.500e-15
BMI	rs4307239	G	A	0.012	0.002	1.470e-11
BMI	rs4421883	C	T	0.010	0.002	1.790e-09
BMI	rs459552	T	A	0.013	0.002	8.360e-12
BMI	rs4609871	T	C	0.022	0.002	6.620e-38
BMI	rs4671328	T	G	0.021	0.002	3.420e-36
BMI	rs4700608	C	T	0.016	0.002	4.320e-20
BMI	rs4721089	T	C	0.017	0.002	5.600e-13
BMI	rs4740619	T	C	0.019	0.002	3.150e-31
BMI	rs4834272	C	T	0.011	0.002	3.460e-10
BMI	rs4841504	C	A	0.017	0.002	5.410e-24
BMI	rs4857968	G	A	0.013	0.002	6.500e-12
BMI	rs487152	A	C	0.011	0.002	2.980e-11
BMI	rs4880341	C	T	0.013	0.002	3.060e-14
BMI	rs4906263	G	C	0.018	0.002	8.110e-23
BMI	rs4973618	G	A	0.015	0.002	1.250e-16
BMI	rs5215	T	C	0.011	0.002	1.220e-11
BMI	rs543874	G	A	0.048	0.002	3.06e-125
BMI	rs561136	C	T	0.019	0.002	1.430e-14
BMI	rs56356382	T	C	0.022	0.002	3.210e-19
BMI	rs57800857	A	C	0.016	0.002	3.490e-15
BMI	rs592483	C	T	0.014	0.002	1.530e-16
BMI	rs59302296	A	T	0.022	0.003	9.120e-12
BMI	rs594821	C	T	0.019	0.003	3.220e-10
BMI	rs615568	G	T	0.010	0.002	3.850e-09
BMI	rs61813324	T	C	0.029	0.003	3.200e-24
BMI	rs61828641	A	G	0.022	0.003	2.490e-13
BMI	rs6265	C	T	0.041	0.002	7.400e-89
BMI	rs6443750	C	T	0.015	0.002	7.250e-13
BMI	rs6451675	G	C	0.014	0.002	2.540e-14
BMI	rs6463489	T	C	0.017	0.003	2.500e-10
BMI	rs6476617	G	A	0.015	0.002	1.530e-17
BMI	rs6477694	C	T	0.013	0.002	6.570e-14
BMI	rs6493498	T	C	0.014	0.002	4.840e-17

BMI	rs650198	C	T	0.014	0.002	6.430e-13
BMI	rs6512302	C	G	0.013	0.002	1.520e-11
BMI	rs6539064	C	G	0.019	0.002	1.140e-23
BMI	rs6545714	G	A	0.019	0.002	4.010e-32
BMI	rs6556301	G	T	0.011	0.002	8.140e-11
BMI	rs6567160	C	T	0.055	0.002	7.82e-184
BMI	rs6591407	C	A	0.012	0.002	3.580e-09
BMI	rs6607337	C	T	0.012	0.002	2.550e-11
BMI	rs6661316	T	C	0.012	0.002	1.720e-13
BMI	rs6690398	A	G	0.013	0.002	1.920e-13
BMI	rs6720868	T	C	0.015	0.002	1.820e-17
BMI	rs6804181	A	T	0.014	0.002	6.470e-10
BMI	rs6804842	G	A	0.014	0.002	7.570e-18
BMI	rs6850421	A	G	0.011	0.002	3.660e-09
BMI	rs6864049	G	A	0.012	0.002	1.480e-13
BMI	rs6890310	G	A	0.012	0.002	3.290e-10
BMI	rs6922607	G	A	0.013	0.002	1.850e-09
BMI	rs6932930	G	A	0.026	0.002	1.180e-40
BMI	rs6962280	G	A	0.014	0.002	3.370e-13
BMI	rs698147	A	G	0.012	0.002	9.670e-12
BMI	rs7024334	T	G	0.014	0.002	4.710e-12
BMI	rs7037266	C	A	0.011	0.002	1.240e-09
BMI	rs704061	C	T	0.014	0.002	8.340e-17
BMI	rs7070670	C	T	0.013	0.002	7.140e-10
BMI	rs7084454	A	G	0.020	0.002	4.510e-27
BMI	rs709400	A	G	0.015	0.002	5.700e-19
BMI	rs7124681	A	C	0.026	0.002	3.960e-55
BMI	rs7138803	A	G	0.030	0.002	3.100e-71
BMI	rs7144011	T	G	0.026	0.002	2.370e-40
BMI	rs7181610	A	T	0.015	0.003	4.500e-09
BMI	rs7206608	G	C	0.013	0.002	1.200e-12
BMI	rs7238896	G	A	0.022	0.003	2.490e-15
BMI	rs7239114	A	G	0.012	0.002	6.240e-13
BMI	rs72673947	G	A	0.022	0.003	4.110e-13
BMI	rs7357754	G	A	0.012	0.002	1.810e-12
BMI	rs740157	A	G	0.012	0.002	4.760e-13
BMI	rs7498665	G	A	0.029	0.002	1.140e-66
BMI	rs7534091	G	A	0.012	0.002	6.650e-11
BMI	rs7561278	T	C	0.017	0.002	4.890e-16
BMI	rs7570446	A	C	0.011	0.002	4.740e-09
BMI	rs7575118	T	C	0.014	0.002	3.330e-09
BMI	rs7588437	G	A	0.017	0.002	2.320e-22
BMI	rs7599312	G	A	0.018	0.002	1.520e-23
BMI	rs7607490	A	G	0.016	0.003	1.210e-09
BMI	rs7616009	G	A	0.016	0.002	4.330e-11
BMI	rs7631156	A	G	0.022	0.002	3.330e-32

BMI	rs765875	C	T	0.013	0.002	1.080e-14
BMI	rs7678054	G	A	0.010	0.002	4.570e-09
BMI	rs76942203	A	G	0.026	0.004	9.080e-11
BMI	rs7713317	G	A	0.017	0.002	1.960e-20
BMI	rs7734385	G	A	0.010	0.002	6.080e-10
BMI	rs77432547	G	A	0.017	0.002	1.440e-15
BMI	rs7769594	T	C	0.016	0.002	2.130e-12
BMI	rs7777084	A	G	0.013	0.002	1.580e-14
BMI	rs7802342	G	T	0.012	0.002	6.230e-11
BMI	rs7826312	C	T	0.011	0.002	3.230e-11
BMI	rs7893571	T	G	0.013	0.002	5.830e-12
BMI	rs7899106	G	A	0.033	0.004	1.720e-18
BMI	rs7903146	C	T	0.018	0.002	1.670e-23
BMI	rs79113395	G	A	0.020	0.002	2.030e-20
BMI	rs7944782	G	T	0.014	0.002	3.610e-17
BMI	rs7975187	G	A	0.014	0.002	3.860e-11
BMI	rs8024806	T	C	0.025	0.004	6.730e-13
BMI	rs8075273	C	A	0.014	0.002	4.670e-14
BMI	rs8122855	A	G	0.014	0.002	4.110e-14
BMI	rs8134638	C	T	0.013	0.002	1.570e-11
BMI	rs816367	G	C	0.011	0.002	1.020e-09
BMI	rs8181823	C	A	0.013	0.002	4.360e-10
BMI	rs845084	A	G	0.014	0.002	3.220e-12
BMI	rs867560	G	C	0.014	0.002	1.240e-15
BMI	rs872281	C	T	0.015	0.002	1.560e-11
BMI	rs889398	C	T	0.020	0.002	3.230e-32
BMI	rs891387	T	C	0.021	0.002	9.260e-35
BMI	rs930295	A	C	0.021	0.002	2.030e-19
BMI	rs9320823	C	T	0.017	0.002	2.070e-21
BMI	rs942066	G	A	0.020	0.002	2.360e-24
BMI	rs946824	T	C	0.020	0.003	4.820e-15
BMI	rs9540493	A	G	0.013	0.002	7.850e-15
BMI	rs9595908	T	C	0.015	0.002	3.730e-20
BMI	rs9603697	T	C	0.013	0.002	1.690e-13
BMI	rs9808302	G	A	0.012	0.002	9.050e-10
BMI	rs9816226	T	A	0.032	0.002	1.450e-50
BMI	rs9818122	C	T	0.023	0.002	3.970e-30
BMI	rs9826775	A	G	0.016	0.002	6.610e-11
BMI	rs9862795	T	A	0.026	0.002	4.430e-44
BMI	rs9937053	A	G	0.072	0.002	1.00e-200
