# Supplementary material for: Body size and composition and risk of site-specific cancers in the UK Biobank and large international consortia: A mendelian randomisation study
Source: PLoS Med. 2021 Jul 29;18(7):e1003706. doi: 10.1371/journal.pmed.1003706 (PMC8320991; doi:10.1371/journal.pmed.1003706)
Supplement: S3 Table — MR, mendelian randomisation; SNP, single nucleotide polymorphism. (PDF) [file pmed.1003706.s007.pdf]

Table S3. Single-nucleotide polymorphisms used as instrumental variables in the multivariable Mendelian randomization analyses of fat mass and fat-free mass indices

				Fat mass index		Fat-free mass index	
SNP	Chromosome	Effect allele	Other allele	Beta	SE	Beta	SE
rs1022523	1	A	G	0.004	0.002	0.007	0.002
rs10798945	1	T	C	0.006	0.002	0.007	0.002
rs111901479	1	A	G	0.000	0.004	0.002	0.003
rs11205303	1	C	T	0.011	0.002	-0.018	0.002
rs11205617	1	A	G	0.017	0.002	0.010	0.002
rs11208779	1	C	G	0.013	0.002	0.008	0.002
rs11240565	1	T	C	0.002	0.002	0.005	0.002
rs11580196	1	G	A	0.000	0.003	0.009	0.002
rs11580836	1	T	C	0.011	0.002	0.006	0.002
rs11803905	1	T	C	0.012	0.002	0.010	0.002
rs12031634	1	A	G	-0.015	0.002	-0.010	0.002
rs12041740	1	A	G	-0.007	0.002	-0.009	0.002
rs12072845	1	A	G	-0.003	0.002	-0.006	0.002
rs12073468	1	T	C	0.007	0.003	0.008	0.002
rs12140153	1	T	G	-0.031	0.004	-0.022	0.003
rs12144626	1	C	T	-0.016	0.002	-0.011	0.002
rs1335055	1	A	G	-0.014	0.002	-0.007	0.002
rs149229890	1	T	G	0.015	0.011	0.008	0.008
rs161799	1	G	A	-0.009	0.002	-0.005	0.002
rs17014332	1	C	T	0.013	0.003	0.010	0.002
rs17363646	1	G	A	0.006	0.003	0.007	0.002
rs1815518	1	T	C	-0.001	0.003	0.003	0.002
rs1952527	1	C	T	-0.003	0.002	-0.006	0.002
rs197372	1	C	T	0.012	0.002	0.009	0.002
rs2181375	1	G	A	0.017	0.002	0.009	0.002
rs2281175	1	C	T	0.007	0.002	0.013	0.002
rs2678204	1	G	T	0.020	0.002	0.017	0.002
rs2803888	1	A	C	-0.002	0.002	-0.003	0.002
rs2815753	1	A	G	0.020	0.002	0.015	0.002
rs28391281	1	C	T	-0.003	0.002	-0.001	0.002
rs284315	1	G	A	-0.006	0.002	-0.012	0.002
rs2885697	1	T	G	0.003	0.002	-0.004	0.002
rs34517439	1	A	C	0.033	0.003	0.025	0.003
rs35804313	1	T	C	-0.002	0.003	0.000	0.002
rs3737992	1	A	G	-0.014	0.003	-0.011	0.002
rs3738449	1	A	G	-0.005	0.002	-0.004	0.002
rs3765351	1	T	C	0.000	0.002	-0.002	0.002
rs3845344	1	T	C	0.015	0.002	0.014	0.002
rs4472800	1	T	G	0.010	0.002	0.012	0.002
rs4657796	1	C	T	-0.014	0.002	-0.007	0.002

rs475591	1	C	T	0.005	0.002	0.007	0.002
rs545608	1	C	G	0.043	0.003	0.033	0.002
rs60077625	1	A	G	0.001	0.002	-0.003	0.002
rs60804050	1	A	G	0.006	0.002	0.010	0.002
rs72660086	1	G	T	0.017	0.003	0.014	0.002
rs75641275	1	C	A	0.021	0.003	0.009	0.002
rs76798800	1	T	G	0.008	0.002	0.020	0.002
rs79113395	1	A	G	-0.018	0.002	-0.012	0.002
rs815341	1	G	A	0.013	0.002	0.010	0.002
rs946197	1	C	G	0.004	0.003	0.009	0.002
rs10170971	2	C	G	-0.001	0.002	0.000	0.002
rs10172196	2	A	G	0.015	0.002	0.003	0.002
rs10203320	2	C	T	0.007	0.002	0.011	0.002
rs1047891	2	A	C	0.006	0.002	0.014	0.002
rs10490530	2	G	A	0.017	0.003	0.011	0.002
rs1064213	2	A	G	0.009	0.002	0.018	0.002
rs10931008	2	C	T	-0.003	0.002	-0.008	0.002
rs11125074	2	A	G	0.001	0.002	0.002	0.002
rs113902512	2	A	G	0.002	0.003	0.009	0.002
rs11545482	2	T	C	-0.014	0.008	-0.027	0.006
rs116072427	2	C	G	-0.014	0.004	-0.007	0.003
rs11678716	2	C	G	0.009	0.004	0.007	0.003
rs11689727	2	A	C	-0.003	0.002	0.008	0.002
rs11695700	2	T	G	0.011	0.002	0.004	0.002
rs12475388	2	A	G	-0.009	0.002	-0.006	0.002
rs12477385	2	T	G	-0.014	0.003	-0.010	0.002
rs1260326	2	C	T	0.004	0.002	0.015	0.002
rs12619348	2	T	C	-0.003	0.003	-0.005	0.002
rs12713004	2	G	A	-0.009	0.002	0.001	0.002
rs13427822	2	G	A	-0.013	0.002	-0.013	0.002
rs13430869	2	T	G	0.002	0.002	0.011	0.002
rs139590892	2	A	G	-0.006	0.003	-0.004	0.002
rs1446585	2	G	A	-0.014	0.003	-0.007	0.002
rs1478575	2	A	T	0.003	0.002	0.008	0.002
rs1542224	2	C	T	0.004	0.002	0.010	0.002
rs17741497	2	A	G	-0.007	0.002	-0.005	0.002
rs1805165	2	A	C	0.001	0.002	0.004	0.002
rs1837367	2	A	G	0.006	0.002	0.004	0.002
rs1840126	2	C	A	-0.012	0.002	-0.010	0.002
rs2140046	2	C	T	0.003	0.002	-0.001	0.002
rs2197563	2	A	G	0.002	0.002	0.002	0.002
rs2289976	2	A	G	0.004	0.002	0.005	0.002
rs2396348	2	T	C	-0.005	0.002	0.007	0.002
rs2861685	2	C	T	-0.017	0.002	-0.011	0.002
rs3116201	2	A	G	0.001	0.004	0.009	0.003
rs343935	2	C	T	0.007	0.003	0.004	0.002

rs35882248	2	T	C	0.016	0.002	0.008	0.002
rs4471907	2	A	G	0.012	0.002	0.005	0.002
rs4482463	2	A	C	-0.028	0.004	-0.018	0.003
rs4549080	2	T	C	0.016	0.002	0.010	0.002
rs4670172	2	A	T	0.010	0.002	0.006	0.002
rs4671328	2	G	T	-0.019	0.002	-0.015	0.002
rs4673617	2	T	C	0.014	0.002	0.008	0.002
rs59985551	2	T	C	-0.001	0.003	0.017	0.002
rs62106258	2	C	T	-0.076	0.005	-0.068	0.004
rs62178054	2	C	T	-0.016	0.002	-0.008	0.002
rs6545714	2	A	G	-0.017	0.002	-0.011	0.002
rs6717858	2	C	T	0.019	0.002	-0.004	0.002
rs71414738	2	T	C	0.003	0.003	0.010	0.002
rs72820274	2	A	G	0.012	0.002	0.010	0.002
rs72885917	2	C	A	-0.003	0.002	-0.015	0.002
rs73966422	2	G	C	0.017	0.003	0.013	0.002
rs7559547	2	T	C	0.041	0.003	0.040	0.002
rs7584391	2	A	G	-0.012	0.003	-0.008	0.002
rs78964719	2	T	G	0.002	0.004	0.003	0.003
rs79883557	2	T	C	0.004	0.004	-0.004	0.003
rs11717749	3	T	C	0.003	0.003	0.007	0.003
rs12330631	3	T	C	-0.013	0.002	-0.004	0.002
rs13062093	3	G	T	0.016	0.002	0.009	0.002
rs1454687	3	G	C	-0.018	0.002	-0.012	0.002
rs17056859	3	A	G	-0.001	0.002	0.001	0.002
rs1881975	3	G	A	0.000	0.003	-0.009	0.002
rs2016469	3	A	G	0.013	0.002	0.009	0.002
rs2270894	3	G	C	-0.003	0.003	0.000	0.002
rs2606228	3	C	A	-0.013	0.002	-0.007	0.002
rs34693680	3	T	C	0.001	0.003	0.005	0.002
rs357501	3	A	G	0.010	0.002	0.010	0.002
rs3749387	3	C	G	0.001	0.002	0.014	0.002
rs4076108	3	T	A	-0.001	0.002	0.005	0.002
rs4627713	3	C	T	0.007	0.007	0.010	0.005
rs4635681	3	G	A	0.008	0.003	0.007	0.002
rs4856720	3	C	G	0.012	0.002	0.010	0.002
rs4858697	3	G	A	0.010	0.002	0.010	0.002
rs509035	3	A	G	-0.006	0.002	0.002	0.002
rs62246314	3	A	G	0.021	0.004	0.011	0.003
rs6445198	3	T	G	-0.014	0.002	-0.012	0.002
rs6762578	3	A	G	0.000	0.003	-0.003	0.002
rs6800021	3	A	G	0.020	0.002	0.022	0.002
rs724016	3	G	A	0.007	0.002	-0.005	0.002
rs73052033	3	C	T	-0.021	0.003	-0.025	0.002
rs73175572	3	G	A	0.004	0.003	0.004	0.003
rs7624428	3	A	T	0.018	0.002	0.014	0.002

rs7628343	3	C	T	-0.002	0.004	0.002	0.003
rs7649970	3	T	C	0.030	0.003	-0.008	0.002
rs8192675	3	C	T	0.014	0.002	0.013	0.002
rs9826759	3	T	C	0.009	0.002	0.015	0.002
rs9861443	3	C	A	0.013	0.002	0.010	0.002
rs9882731	3	C	G	-0.001	0.002	0.002	0.002
rs9968060	3	T	C	0.012	0.002	0.006	0.002
rs10024321	4	C	G	-0.001	0.002	-0.001	0.002
rs10938398	4	A	G	0.028	0.002	0.018	0.002
rs11098675	4	G	A	-0.009	0.003	-0.011	0.002
rs111632154	4	C	T	-0.005	0.005	-0.008	0.004
rs115574684	4	A	T	0.013	0.006	0.017	0.004
rs1229984	4	C	T	0.045	0.007	0.024	0.005
rs13124829	4	G	A	0.014	0.002	0.010	0.002
rs13148166	4	G	T	0.001	0.002	-0.003	0.002
rs1383723	4	T	A	-0.016	0.003	-0.008	0.002
rs1472852	4	A	C	0.013	0.003	0.005	0.002
rs1724551	4	A	G	-0.015	0.002	-0.011	0.002
rs17454369	4	C	G	0.003	0.005	0.015	0.004
rs1878528	4	G	A	0.005	0.002	-0.007	0.002
rs2102278	4	G	A	0.010	0.002	0.004	0.002
rs2592831	4	C	T	0.005	0.002	0.003	0.002
rs2869950	4	T	C	-0.011	0.002	0.000	0.002
rs2968669	4	G	C	0.004	0.002	0.005	0.002
rs34028346	4	A	G	0.010	0.003	0.009	0.002
rs35851183	4	G	A	0.012	0.002	0.008	0.002
rs4235012	4	C	T	0.009	0.002	0.012	0.002
rs4240326	4	G	A	-0.007	0.002	-0.005	0.002
rs4864201	4	C	T	-0.013	0.002	-0.008	0.002
rs62275882	4	A	G	0.000	0.003	-0.007	0.002
rs6821305	4	C	A	0.006	0.002	0.005	0.002
rs6840236	4	C	T	0.012	0.002	-0.002	0.002
rs72703414	4	G	A	-0.001	0.004	0.001	0.003
rs73213484	4	T	A	-0.019	0.003	-0.010	0.002
rs750090	4	C	T	-0.011	0.002	-0.007	0.002
rs981002	4	A	T	0.002	0.002	0.000	0.002
rs9985795	4	C	T	-0.005	0.002	-0.005	0.002
rs115912456	5	G	A	-0.021	0.005	0.033	0.004
rs11951673	5	T	C	-0.007	0.002	-0.010	0.002
rs11958027	5	G	A	0.014	0.002	0.009	0.002
rs1477290	5	C	T	0.031	0.003	0.018	0.002
rs1503527	5	T	C	0.015	0.002	0.009	0.002
rs153560	5	A	G	0.008	0.002	0.005	0.002
rs1582931	5	A	G	-0.009	0.002	-0.012	0.002
rs16869017	5	T	C	0.005	0.004	0.007	0.003
rs183041	5	A	G	-0.002	0.002	-0.004	0.002

rs2118663	5	C	T	-0.011	0.003	-0.007	0.002
rs2307111	5	C	T	-0.025	0.002	-0.017	0.002
rs247008	5	G	A	0.006	0.002	0.003	0.002
rs252758	5	A	T	-0.013	0.002	-0.007	0.002
rs252937	5	G	C	-0.002	0.002	-0.007	0.002
rs254963	5	G	A	0.002	0.002	0.002	0.002
rs28636067	5	A	G	0.012	0.003	0.002	0.002
rs292168	5	G	A	0.003	0.002	-0.004	0.002
rs299370	5	C	T	0.006	0.002	0.006	0.002
rs33967909	5	A	G	0.006	0.003	0.008	0.002
rs396755	5	G	C	-0.013	0.002	-0.006	0.002
rs40071	5	C	T	-0.022	0.003	-0.018	0.002
rs4073717	5	T	G	-0.005	0.003	-0.002	0.002
rs4282339	5	A	G	0.001	0.003	-0.002	0.002
rs4865956	5	A	T	0.008	0.002	0.004	0.002
rs55681913	5	C	T	-0.002	0.004	0.010	0.003
rs55758152	5	A	G	0.000	0.002	-0.002	0.002
rs6235	5	G	C	0.011	0.002	0.009	0.002
rs67913249	5	G	C	-0.013	0.002	-0.007	0.002
rs6861649	5	C	T	0.014	0.002	0.005	0.002
rs6873192	5	G	A	-0.007	0.002	-0.007	0.002
rs6874142	5	G	T	0.005	0.004	0.003	0.003
rs6898357	5	G	A	-0.012	0.002	-0.007	0.002
rs7442885	5	G	C	-0.021	0.003	-0.016	0.002
rs7707394	5	A	G	-0.017	0.002	-0.011	0.002
rs7709645	5	C	G	-0.004	0.002	-0.008	0.002
rs7730885	5	G	A	0.012	0.002	0.009	0.002
rs7731023	5	G	A	0.000	0.002	0.005	0.002
rs9327336	5	C	T	0.001	0.002	0.000	0.002
rs10499014	6	G	C	-0.017	0.002	-0.009	0.002
rs10947793	6	G	A	-0.014	0.002	-0.007	0.002
rs12193797	6	G	A	-0.010	0.003	-0.013	0.002
rs12209223	6	A	C	0.000	0.004	-0.006	0.003
rs12216497	6	T	C	-0.004	0.002	-0.011	0.002
rs1591806	6	G	A	-0.011	0.002	0.000	0.002
rs2073272	6	A	G	-0.007	0.002	-0.008	0.002
rs2395617	6	C	A	0.006	0.003	0.007	0.002
rs2457982	6	A	G	-0.004	0.002	0.003	0.002
rs2482398	6	A	C	-0.014	0.002	-0.010	0.002
rs2499468	6	A	C	0.013	0.002	0.007	0.002
rs2503756	6	T	C	0.000	0.002	-0.002	0.002
rs2744965	6	T	C	0.030	0.003	0.013	0.002
rs2856666	6	T	G	-0.001	0.002	-0.006	0.002
rs34045288	6	T	C	0.020	0.002	0.016	0.002
rs3778157	6	C	T	0.013	0.003	0.012	0.002
rs3798519	6	C	A	0.032	0.003	0.027	0.002

rs3853252	6	A	G	0.005	0.002	0.002	0.002
rs3864311	6	G	T	-0.011	0.002	-0.006	0.002
rs412522	6	C	T	0.009	0.002	0.005	0.002
rs41271299	6	T	C	0.002	0.005	-0.015	0.004
rs599004	6	T	C	-0.006	0.002	-0.009	0.002
rs6570509	6	T	G	0.001	0.002	0.013	0.002
rs6902789	6	A	G	0.002	0.002	-0.013	0.002
rs730536	6	C	G	-0.005	0.002	-0.007	0.002
rs765875	6	T	C	-0.013	0.002	-0.007	0.002
rs768023	6	A	G	0.014	0.002	0.011	0.002
rs7740107	6	A	T	-0.006	0.002	-0.011	0.002
rs79266482	6	T	C	0.011	0.002	0.009	0.002
rs9257319	6	G	T	-0.009	0.003	-0.008	0.002
rs9320823	6	C	T	0.020	0.002	0.008	0.002
rs9343977	6	T	C	0.000	0.002	-0.001	0.002
rs9379084	6	A	G	0.005	0.003	0.007	0.003
rs9379130	6	C	G	0.000	0.002	0.000	0.002
rs9381350	6	A	T	-0.002	0.002	0.004	0.002
rs9480947	6	G	C	-0.002	0.002	-0.005	0.002
rs10236214	7	T	C	0.008	0.002	0.014	0.002
rs10237306	7	T	G	0.013	0.002	0.007	0.002
rs10257870	7	A	C	0.001	0.004	0.010	0.003
rs10260993	7	G	T	0.001	0.003	-0.004	0.002
rs10953112	7	C	T	0.018	0.003	0.021	0.002
rs111964059	7	A	T	0.008	0.004	0.007	0.003
rs11764337	7	T	C	-0.017	0.003	-0.012	0.002
rs12540011	7	A	G	0.003	0.002	0.009	0.002
rs13247154	7	A	G	0.007	0.002	0.009	0.002
rs143986132	7	G	A	-0.005	0.008	-0.009	0.006
rs17157112	7	G	T	0.002	0.002	0.004	0.002
rs2289379	7	T	C	-0.017	0.002	-0.012	0.002
rs2396625	7	A	T	-0.017	0.002	-0.012	0.002
rs2533879	7	A	G	-0.007	0.002	0.004	0.002
rs28457808	7	G	C	-0.019	0.003	-0.014	0.002
rs2866719	7	T	C	0.010	0.002	0.007	0.002
rs34776209	7	T	C	0.003	0.002	0.000	0.002
rs3800963	7	A	G	-0.002	0.002	-0.006	0.002
rs42044	7	G	T	0.003	0.002	0.001	0.002
rs508347	7	C	T	-0.003	0.002	0.008	0.002
rs58862095	7	T	C	-0.020	0.002	-0.016	0.002
rs62621812	7	A	G	0.013	0.008	0.048	0.006
rs6973656	7	G	A	0.014	0.002	0.005	0.002
rs6975015	7	A	G	0.009	0.003	0.012	0.003
rs723149	7	G	A	0.002	0.002	-0.002	0.002
rs7781964	7	A	G	-0.001	0.003	0.005	0.002
rs836519	7	T	C	0.012	0.003	0.008	0.002

rs10954772	8	C	T	-0.017	0.002	-0.008	0.002
rs10958683	8	G	C	0.000	0.003	-0.009	0.002
rs11782341	8	G	A	0.015	0.003	0.005	0.002
rs11783086	8	C	T	0.000	0.002	-0.001	0.002
rs11786089	8	G	A	0.013	0.002	0.002	0.002
rs12682601	8	A	C	-0.010	0.002	-0.011	0.002
rs13264909	8	T	A	-0.010	0.002	-0.007	0.002
rs1452822	8	A	T	0.000	0.002	0.003	0.002
rs16892552	8	G	A	0.004	0.002	-0.001	0.002
rs1910252	8	T	C	0.004	0.003	0.009	0.002
rs2134963	8	A	C	-0.003	0.002	-0.016	0.002
rs2142331	8	T	C	-0.005	0.002	-0.007	0.002
rs2277138	8	C	T	-0.007	0.002	-0.004	0.002
rs2280940	8	T	C	0.006	0.002	0.003	0.002
rs2979655	8	G	T	0.014	0.003	0.009	0.002
rs310302	8	A	G	-0.002	0.002	-0.002	0.002
rs4876611	8	G	A	0.017	0.002	0.005	0.002
rs59104534	8	T	C	0.011	0.002	0.006	0.002
rs61729527	8	T	C	-0.010	0.005	-0.014	0.004
rs62499696	8	C	G	0.013	0.002	0.008	0.002
rs62515437	8	T	G	-0.003	0.003	0.000	0.002
rs72656010	8	C	T	-0.003	0.003	-0.011	0.002
rs7460093	8	A	G	0.008	0.002	0.005	0.002
rs7815955	8	T	A	0.003	0.003	0.006	0.002
rs7845090	8	A	G	-0.022	0.002	-0.011	0.002
rs9314420	8	G	A	0.001	0.002	0.001	0.002
rs931874	8	T	C	0.002	0.002	0.004	0.002
rs10746837	9	A	G	-0.001	0.002	0.003	0.002
rs10756714	9	G	A	-0.021	0.002	-0.008	0.002
rs10756798	9	T	C	-0.016	0.002	-0.008	0.002
rs10820852	9	A	C	-0.010	0.002	-0.010	0.002
rs10982888	9	A	T	0.011	0.003	-0.004	0.003
rs10990621	9	G	A	-0.012	0.003	-0.003	0.002
rs1111818	9	C	G	0.007	0.002	0.006	0.002
rs113457986	9	G	A	0.028	0.009	0.026	0.007
rs12339822	9	G	A	0.015	0.002	0.007	0.002
rs12347137	9	C	A	0.002	0.003	-0.007	0.002
rs13294021	9	A	G	-0.002	0.002	-0.006	0.002
rs141403611	9	G	C	-0.006	0.004	-0.006	0.003
rs17218879	9	G	C	0.012	0.002	0.007	0.002
rs17770336	9	T	C	0.020	0.002	0.017	0.002
rs1927635	9	C	T	0.004	0.002	0.004	0.002
rs2274116	9	T	C	0.001	0.002	0.001	0.002
rs28457693	9	G	A	-0.003	0.003	0.011	0.003
rs35436119	9	A	G	0.001	0.004	0.004	0.003
rs4503172	9	T	C	-0.014	0.002	-0.007	0.002

rs7047694	9	A	G	0.012	0.002	0.010	0.002
rs10787738	10	T	C	0.017	0.002	0.010	0.002
rs10827289	10	T	C	-0.011	0.002	-0.008	0.002
rs10883560	10	G	C	0.006	0.002	0.010	0.002
rs10887578	10	C	G	0.012	0.002	0.006	0.002
rs10995366	10	A	G	-0.001	0.002	-0.003	0.002
rs10999460	10	T	C	0.012	0.002	-0.011	0.002
rs11012732	10	G	A	0.022	0.002	0.011	0.002
rs11013045	10	G	T	-0.005	0.002	-0.007	0.002
rs11014285	10	A	G	0.000	0.003	0.007	0.002
rs11245450	10	A	G	-0.011	0.002	-0.010	0.002
rs11594322	10	C	A	0.003	0.003	-0.001	0.002
rs117543413	10	T	C	-0.005	0.008	-0.008	0.006
rs12218394	10	C	G	0.009	0.002	0.001	0.002
rs12254441	10	T	C	-0.013	0.002	-0.006	0.002
rs2172131	10	C	T	-0.015	0.002	-0.008	0.002
rs2439823	10	G	A	0.017	0.002	0.012	0.002
rs2476998	10	T	C	0.008	0.002	0.010	0.002
rs4752689	10	A	G	0.004	0.002	0.004	0.002
rs4980067	10	A	C	-0.002	0.002	0.002	0.002
rs61862463	10	G	A	0.016	0.007	0.018	0.005
rs61871615	10	T	C	-0.022	0.004	-0.013	0.003
rs662115	10	T	C	-0.004	0.002	0.001	0.002
rs7077783	10	T	C	-0.006	0.003	-0.007	0.002
rs7080472	10	T	G	-0.009	0.002	0.018	0.002
rs7094644	10	A	G	0.012	0.002	0.008	0.002
rs7097872	10	T	C	0.009	0.002	0.008	0.002
rs71484923	10	G	T	0.013	0.003	0.010	0.002
rs7910087	10	C	T	0.000	0.002	-0.011	0.002
rs10128597	11	A	G	-0.015	0.002	-0.007	0.002
rs10767735	11	C	G	-0.008	0.002	-0.010	0.002
rs11023199	11	G	A	-0.009	0.002	-0.002	0.002
rs11030119	11	A	G	0.027	0.002	0.021	0.002
rs11217863	11	A	G	-0.005	0.003	-0.001	0.003
rs11233117	11	G	C	-0.003	0.002	-0.003	0.002
rs11603783	11	C	T	0.014	0.002	0.005	0.002
rs11824092	11	C	T	0.013	0.002	0.007	0.002
rs1222219	11	C	G	-0.012	0.003	-0.010	0.002
rs1228024	11	A	C	0.004	0.002	0.000	0.002
rs1783541	11	T	C	0.018	0.003	0.012	0.002
rs1789164	11	G	C	0.008	0.002	0.010	0.002
rs1881505	11	C	T	-0.028	0.005	-0.012	0.004
rs34292685	11	T	C	-0.017	0.003	-0.008	0.002
rs35506085	11	A	G	-0.005	0.003	-0.007	0.002
rs57153895	11	G	A	0.007	0.002	-0.010	0.002
rs57635800	11	A	G	0.019	0.002	0.014	0.002

rs607472	11	G	C	0.003	0.002	0.004	0.002
rs61903695	11	G	A	0.015	0.002	0.010	0.002
rs7129320	11	A	G	-0.003	0.003	-0.013	0.002
rs72906282	11	G	T	0.045	0.009	0.023	0.007
rs73041988	11	G	T	-0.019	0.003	-0.007	0.002
rs74048171	11	A	C	-0.005	0.002	-0.005	0.002
rs74565893	11	T	C	-0.010	0.010	-0.003	0.007
rs7933085	11	G	A	0.015	0.002	0.009	0.002
rs7952436	11	T	C	-0.005	0.004	0.017	0.003
rs10748128	12	T	G	-0.002	0.002	-0.005	0.002
rs10770705	12	C	A	0.000	0.002	0.002	0.002
rs10843139	12	G	T	0.000	0.002	0.007	0.002
rs10847415	12	C	T	0.016	0.002	0.005	0.002
rs11065015	12	T	C	-0.029	0.007	-0.014	0.005
rs11065979	12	T	C	-0.006	0.002	-0.010	0.002
rs12578952	12	G	A	-0.015	0.002	-0.014	0.002
rs12813149	12	A	G	-0.018	0.003	-0.009	0.002
rs1351394	12	C	T	0.000	0.002	-0.003	0.002
rs1716162	12	A	T	0.005	0.003	0.002	0.002
rs1964599	12	T	C	-0.016	0.002	0.001	0.002
rs2229840	12	T	C	0.008	0.003	0.011	0.002
rs2287214	12	G	A	0.015	0.002	0.001	0.002
rs2374947	12	G	A	0.015	0.003	0.009	0.002
rs2733287	12	C	A	0.013	0.002	0.009	0.002
rs2897968	12	A	G	0.001	0.002	0.011	0.002
rs2900208	12	A	C	-0.002	0.002	0.001	0.002
rs310796	12	T	G	0.004	0.002	0.002	0.002
rs324010	12	A	G	-0.001	0.002	-0.003	0.002
rs35756741	12	T	C	0.007	0.004	-0.001	0.003
rs3730071	12	A	C	-0.024	0.006	-0.010	0.005
rs3759094	12	T	C	-0.011	0.002	-0.011	0.002
rs4980826	12	A	C	0.001	0.002	-0.004	0.002
rs55726687	12	A	G	0.017	0.003	0.015	0.002
rs59066241	12	G	T	0.018	0.003	0.008	0.003
rs61649432	12	T	C	0.013	0.002	0.008	0.002
rs61941043	12	T	A	0.019	0.011	0.025	0.008
rs61954257	12	G	A	0.011	0.002	0.006	0.002
rs658957	12	C	T	-0.005	0.003	-0.006	0.002
rs67551338	12	T	C	-0.006	0.005	0.017	0.003
rs7132908	12	A	G	0.024	0.002	0.017	0.002
rs7134283	12	A	G	-0.004	0.002	-0.004	0.002
rs7305516	12	G	A	-0.002	0.002	0.000	0.002
rs75412871	12	T	C	-0.026	0.005	-0.011	0.004
rs76895963	12	G	T	0.027	0.008	0.032	0.006
rs78812993	12	C	G	-0.001	0.005	-0.011	0.004
rs7977788	12	A	G	-0.001	0.003	0.002	0.002

rs10507483	13	C	T	0.017	0.003	0.012	0.002
rs1218824	13	A	G	0.012	0.002	0.009	0.002
rs146851424	13	C	A	0.005	0.007	0.001	0.006
rs1887855	13	C	T	-0.001	0.002	-0.005	0.002
rs1928496	13	T	C	0.014	0.002	0.009	0.002
rs2479958	13	G	A	-0.016	0.002	-0.008	0.002
rs2490637	13	A	C	-0.003	0.004	0.001	0.003
rs3118915	13	T	C	0.002	0.003	-0.008	0.002
rs3783256	13	C	T	0.000	0.002	-0.003	0.002
rs3818416	13	C	A	0.008	0.003	0.012	0.002
rs4477562	13	T	C	0.024	0.003	0.018	0.002
rs748457	13	A	G	0.010	0.002	0.011	0.002
rs7987928	13	A	G	-0.018	0.003	-0.006	0.002
rs7994814	13	T	C	-0.002	0.002	0.010	0.002
rs9317002	13	A	C	0.011	0.002	0.012	0.002
rs9515455	13	A	G	0.014	0.002	0.009	0.002
rs9540493	13	G	A	-0.012	0.002	-0.010	0.002
rs9584855	13	G	T	-0.015	0.002	-0.005	0.002
rs10144067	14	T	C	0.016	0.002	0.013	0.002
rs10483727	14	C	T	-0.001	0.002	-0.004	0.002
rs11158820	14	G	A	0.001	0.002	-0.010	0.002
rs112957890	14	G	A	0.007	0.002	0.002	0.002
rs1285990	14	T	C	0.010	0.002	0.013	0.002
rs12879423	14	G	A	0.016	0.002	0.018	0.002
rs12887636	14	G	T	-0.013	0.002	-0.008	0.002
rs12889702	14	C	A	0.010	0.002	0.008	0.002
rs1491905	14	C	T	-0.014	0.002	-0.012	0.002
rs17197114	14	C	T	0.004	0.003	0.003	0.002
rs217672	14	C	A	0.015	0.002	0.009	0.002
rs2296316	14	C	T	0.004	0.002	0.002	0.002
rs28929474	14	T	C	-0.010	0.008	-0.047	0.006
rs3212260	14	T	A	0.003	0.002	0.001	0.002
rs35233301	14	G	A	0.000	0.002	0.005	0.002
rs36100359	14	A	G	0.005	0.003	-0.005	0.002
rs3803286	14	G	A	-0.017	0.002	-0.012	0.002
rs4900471	14	A	G	-0.010	0.002	-0.007	0.002
rs7141420	14	T	C	0.018	0.002	0.011	0.002
rs7148516	14	A	T	-0.017	0.002	-0.014	0.002
rs72699866	14	A	G	-0.009	0.003	0.014	0.002
rs8007058	14	A	G	0.008	0.003	0.011	0.002
rs8007644	14	A	G	0.005	0.002	0.006	0.002
rs9788443	14	C	T	0.005	0.005	0.005	0.004
rs11855017	15	A	C	0.009	0.003	0.012	0.002
rs11857221	15	A	C	0.014	0.002	0.007	0.002
rs12906197	15	T	C	-0.003	0.002	-0.007	0.002
rs1521624	15	A	C	-0.011	0.002	-0.011	0.002

rs1573891	15	C	G	0.005	0.003	-0.003	0.002
rs16942324	15	A	C	0.010	0.007	0.027	0.005
rs16964211	15	A	G	0.004	0.005	0.003	0.004
rs1879529	15	T	G	-0.004	0.002	0.023	0.002
rs2455561	15	G	T	0.003	0.002	-0.007	0.002
rs28413009	15	T	C	-0.013	0.004	-0.008	0.003
rs35697691	15	G	C	0.025	0.004	0.008	0.003
rs35874463	15	G	A	0.004	0.005	-0.019	0.003
rs3784699	15	C	T	0.021	0.002	0.014	0.002
rs3809570	15	A	C	-0.002	0.003	0.006	0.002
rs5742915	15	C	T	-0.011	0.002	-0.006	0.002
rs62025854	15	T	G	-0.013	0.002	0.023	0.002
rs6598540	15	G	A	-0.013	0.002	-0.007	0.002
rs7171864	15	A	G	0.014	0.002	0.004	0.002
rs72755233	15	A	G	-0.001	0.003	0.031	0.003
rs933807	15	C	G	0.003	0.002	-0.003	0.002
rs10775348	16	G	A	0.001	0.002	0.000	0.002
rs12103006	16	G	A	0.012	0.002	0.002	0.002
rs2411453	16	G	T	-0.029	0.002	-0.012	0.002
rs2539999	16	C	T	0.006	0.002	0.008	0.002
rs2917705	16	A	G	-0.015	0.003	-0.010	0.002
rs30235	16	T	C	-0.003	0.002	0.000	0.002
rs3814877	16	T	G	0.017	0.002	0.017	0.002
rs4782286	16	A	G	-0.012	0.003	-0.019	0.002
rs4985148	16	C	A	-0.008	0.002	-0.006	0.002
rs55872725	16	T	C	0.060	0.002	0.053	0.002
rs71385734	16	G	T	-0.012	0.003	-0.008	0.002
rs7190477	16	A	C	0.004	0.002	0.006	0.002
rs7193783	16	C	A	-0.013	0.002	-0.005	0.002
rs7205337	16	G	A	0.002	0.003	0.001	0.002
rs72801843	16	A	T	0.003	0.002	0.004	0.002
rs77392989	16	C	T	-0.011	0.003	-0.006	0.002
rs78818722	16	T	C	0.006	0.004	0.015	0.003
rs8059189	16	A	G	0.003	0.002	-0.009	0.002
rs862320	16	T	C	-0.020	0.002	-0.013	0.002
rs879620	16	T	C	0.022	0.002	0.016	0.002
rs9925273	16	G	A	-0.010	0.003	-0.007	0.002
rs11150745	17	G	A	-0.020	0.002	-0.015	0.002
rs113866544	17	C	T	0.033	0.004	0.017	0.003
rs11655578	17	T	G	0.003	0.003	0.004	0.002
rs11656758	17	G	A	0.014	0.002	0.001	0.002
rs11657325	17	A	G	0.005	0.002	-0.004	0.002
rs11658134	17	A	G	-0.006	0.002	-0.005	0.002
rs12452505	17	G	C	0.005	0.003	0.002	0.002
rs2005172	17	C	A	-0.009	0.002	0.003	0.002
rs216193	17	G	A	-0.008	0.002	-0.012	0.002

rs3110496	17	G	A	0.001	0.002	0.000	0.002
rs34966008	17	T	C	-0.016	0.002	-0.011	0.002
rs36000545	17	G	A	-0.007	0.002	-0.009	0.002
rs4790841	17	T	C	-0.029	0.003	-0.016	0.002
rs4794222	17	G	A	-0.009	0.002	-0.003	0.002
rs55831773	17	T	C	-0.006	0.003	-0.007	0.002
rs56288810	17	G	A	0.014	0.003	0.008	0.002
rs62070645	17	A	C	-0.004	0.002	0.000	0.002
rs7214743	17	G	A	0.009	0.002	0.007	0.002
rs72828807	17	A	G	0.002	0.002	0.003	0.002
rs78378222	17	G	T	0.014	0.010	0.056	0.008
rs80135947	17	C	A	0.023	0.003	0.005	0.002
rs9915532	17	G	A	-0.007	0.003	-0.001	0.002
rs1552234	18	A	G	0.012	0.002	0.010	0.002
rs35710322	18	C	T	-0.003	0.002	-0.003	0.002
rs4800670	18	C	G	-0.003	0.002	-0.005	0.002
rs57126421	18	G	A	-0.008	0.003	-0.006	0.002
rs57636386	18	C	T	-0.035	0.004	-0.025	0.003
rs6505781	18	C	G	-0.003	0.002	-0.003	0.002
rs66922415	18	G	A	0.042	0.003	0.042	0.002
rs7229351	18	A	G	-0.002	0.002	-0.007	0.002
rs7235010	18	A	G	0.003	0.003	-0.004	0.002
rs7238896	18	G	A	0.020	0.003	0.014	0.002
rs74494415	18	T	C	-0.003	0.006	-0.019	0.004
rs8087074	18	T	G	0.013	0.002	0.007	0.002
rs9951619	18	G	T	0.013	0.003	0.012	0.002
rs9960619	18	T	C	-0.001	0.002	0.004	0.002
rs9964122	18	G	A	0.014	0.002	0.006	0.002
rs10423928	19	A	T	-0.030	0.003	-0.019	0.002
rs1043413	19	G	C	0.002	0.002	-0.003	0.002
rs111640872	19	C	G	0.017	0.002	0.011	0.002
rs11666480	19	G	C	0.014	0.002	0.011	0.002
rs11667280	19	G	C	-0.010	0.003	-0.012	0.002
rs11880992	19	A	G	0.000	0.002	0.000	0.002
rs12459368	19	G	A	-0.016	0.002	-0.009	0.002
rs12610925	19	G	A	0.015	0.002	-0.002	0.002
rs147110934	19	T	G	0.020	0.007	0.012	0.005
rs2305758	19	T	C	-0.004	0.002	0.005	0.002
rs2602713	19	C	A	-0.003	0.002	0.001	0.002
rs281385	19	G	A	-0.005	0.003	-0.011	0.003
rs3810291	19	A	G	0.019	0.002	0.023	0.002
rs3843751	19	T	C	-0.003	0.002	-0.004	0.002
rs429358	19	C	T	-0.022	0.003	-0.012	0.002
rs55714539	19	C	A	0.020	0.002	0.007	0.002
rs58857770	19	G	C	-0.003	0.002	-0.002	0.002
rs62621197	19	T	C	-0.010	0.006	0.035	0.004

rs7250927	19	C	T	-0.001	0.002	-0.001	0.002
rs72976986	19	A	G	-0.019	0.003	-0.012	0.002
rs8112818	19	G	A	-0.017	0.002	-0.011	0.002
rs10485622	20	G	A	-0.016	0.003	-0.016	0.002
rs1056441	20	C	T	0.013	0.002	0.001	0.002
rs11474838	20	G	T	0.014	0.002	0.002	0.002
rs116165844	20	T	G	-0.007	0.003	-0.010	0.002
rs1407031	20	T	C	0.003	0.002	0.008	0.002
rs143384	20	G	A	-0.004	0.002	0.000	0.002
rs17265513	20	C	T	0.011	0.003	-0.005	0.002
rs2252720	20	T	C	0.001	0.002	-0.008	0.002
rs34879158	20	C	A	-0.005	0.002	-0.005	0.002
rs35276559	20	T	C	-0.005	0.002	-0.005	0.002
rs4341996	20	C	A	-0.011	0.003	-0.009	0.002
rs6029180	20	G	A	0.010	0.002	0.004	0.002
rs6032233	20	C	T	0.004	0.003	0.005	0.002
rs6081869	20	G	T	0.002	0.002	-0.005	0.002
rs6085659	20	G	A	-0.001	0.002	-0.001	0.002
rs6142059	20	C	T	0.011	0.002	0.011	0.002
rs73619441	20	G	T	-0.014	0.003	-0.013	0.002
rs8118253	20	A	T	0.016	0.003	0.009	0.002
rs8123912	20	C	G	-0.013	0.003	-0.006	0.002
rs2230033	21	A	G	-0.001	0.002	-0.002	0.002
rs4819021	21	C	T	-0.012	0.002	-0.010	0.002
rs11538	22	G	A	0.013	0.003	0.007	0.002
rs13056506	22	T	G	-0.015	0.002	0.004	0.002
rs35665085	22	A	G	-0.007	0.005	-0.011	0.004
rs41311445	22	C	A	-0.009	0.004	-0.013	0.003
rs4680	22	A	G	0.008	0.002	0.009	0.002
rs5752989	22	A	G	-0.003	0.002	-0.005	0.002
