# Supplementary material for: Body size and composition and risk of site-specific cancers in the UK Biobank and large international consortia: A mendelian randomisation study
Source: PLoS Med. 2021 Jul 29;18(7):e1003706. doi: 10.1371/journal.pmed.1003706 (PMC8320991; doi:10.1371/journal.pmed.1003706)
Supplement: S4 Table — (PDF) [file pmed.1003706.s008.pdf]

Table S4. Single nucleotide polymorphisms used in the analyses of height

Exposure	SNP	Effect allele	Other allele	Beta	SE	*P* value
Height	rs10119624	A	G	0.026	0.003	1.20e-16
Height	rs10131337	T	C	0.027	0.004	5.60e-13
Height	rs10152739	T	A	0.023	0.003	3.10e-11
Height	rs10283100	G	A	0.054	0.009	2.50e-10
Height	rs1036477	A	G	0.032	0.005	2.80e-11
Height	rs1036821	G	A	0.047	0.004	2.80e-38
Height	rs1047014	C	T	0.033	0.004	7.50e-20
Height	rs1074683	C	G	0.047	0.003	0
Height	rs10748128	T	G	0.038	0.003	4.60e-29
Height	rs10767838	A	G	0.025	0.003	1.80e-14
Height	rs10770705	A	C	0.030	0.003	4.80e-22
Height	rs10794175	T	G	0.021	0.003	4.00e-12
Height	rs10843390	T	C	0.025	0.003	4.80e-15
Height	rs10863936	G	A	0.021	0.003	9.00e-13
Height	rs10880969	C	T	0.023	0.003	1.10e-12
Height	rs10948222	C	T	0.032	0.003	8.70e-22
Height	rs10990303	T	C	0.032	0.004	1.40e-19
Height	rs10995319	T	C	0.019	0.003	2.30e-08
Height	rs10997979	G	A	0.018	0.003	3.50e-10
Height	rs11049611	C	T	0.037	0.003	6.30e-30
Height	rs11057552	A	T	0.023	0.004	6.00e-09
Height	rs1113765	G	A	0.025	0.004	1.10e-10
Height	rs11144688	G	A	0.064	0.006	5.90e-24
Height	rs11152213	C	A	0.025	0.004	9.20e-13
Height	rs11221442	G	C	0.027	0.004	3.00e-14
Height	rs11236294	T	G	0.018	0.003	4.50e-08
Height	rs1155939	A	C	0.042	0.003	0
Height	rs11612228	T	C	0.020	0.003	3.70e-10
Height	rs11616380	T	G	0.020	0.003	1.20e-09
Height	rs11624136	A	G	0.017	0.003	3.30e-09
Height	rs11642612	C	A	0.017	0.003	3.20e-08
Height	rs11648796	G	A	0.034	0.004	7.70e-19
Height	rs11659752	T	G	0.025	0.003	2.10e-13
Height	rs11684404	C	T	0.032	0.003	2.30e-25
Height	rs1171615	C	T	0.022	0.004	4.50e-09
Height	rs11722554	G	A	0.059	0.010	2.10e-09
Height	rs11799609	T	G	0.026	0.004	7.00e-10
Height	rs11855014	G	A	0.022	0.003	1.40e-10
Height	rs11867479	T	C	0.025	0.003	2.00e-15
Height	rs11880124	A	G	0.041	0.005	4.00e-14
Height	rs11880992	A	G	0.032	0.003	1.10e-26
Height	rs1190545	C	G	0.024	0.003	2.40e-13

Height	rs1199734	G	T	0.021	0.004	4.00e-08
Height	rs12153391	C	A	0.024	0.004	1.00e-10
Height	rs12190423	G	C	0.016	0.003	4.30e-08
Height	rs12214804	C	T	0.087	0.006	0
Height	rs12228415	G	A	0.017	0.003	2.70e-08
Height	rs12323101	A	G	0.021	0.003	2.40e-12
Height	rs12330322	C	T	0.034	0.004	3.50e-22
Height	rs12435366	C	T	0.023	0.004	3.70e-11
Height	rs1244981	A	G	0.025	0.004	5.30e-10
Height	rs12470505	T	G	0.046	0.005	4.00e-20
Height	rs12513181	C	A	0.019	0.003	2.10e-08
Height	rs12538407	A	G	0.043	0.003	1.00e-35
Height	rs12615742	T	C	0.021	0.004	7.00e-09
Height	rs12639764	T	C	0.027	0.003	5.00e-20
Height	rs12693589	C	T	0.022	0.003	5.50e-11
Height	rs12779328	C	T	0.028	0.003	1.50e-17
Height	rs12882130	C	G	0.024	0.003	2.90e-14
Height	rs13006748	C	G	0.023	0.003	1.00e-11
Height	rs13078528	A	G	0.046	0.006	7.40e-13
Height	rs13150868	T	G	0.018	0.003	1.20e-09
Height	rs13177718	C	T	0.054	0.006	5.40e-19
Height	rs1321666	C	T	0.017	0.003	3.50e-08
Height	rs13388725	G	A	0.018	0.003	2.40e-09
Height	rs13416119	A	G	0.029	0.005	4.90e-09
Height	rs1346490	A	C	0.018	0.003	4.60e-08
Height	rs1401795	A	G	0.030	0.003	5.00e-25
Height	rs14062	G	A	0.018	0.003	1.60e-08
Height	rs1415701	G	A	0.031	0.004	2.50e-16
Height	rs143384	G	A	0.063	0.004	0
Height	rs1544196	G	A	0.019	0.004	2.80e-08
Height	rs1546391	G	C	0.042	0.006	2.50e-12
Height	rs1550162	G	A	0.024	0.003	2.90e-14
Height	rs1552173	C	T	0.018	0.003	2.00e-10
Height	rs1562975	A	G	0.025	0.003	4.00e-15
Height	rs1576900	G	A	0.019	0.003	6.50e-09
Height	rs1614303	T	G	0.023	0.004	1.50e-09
Height	rs165189	G	A	0.031	0.005	2.70e-11
Height	rs1659127	A	G	0.030	0.003	1.20e-19
Height	rs1681630	T	C	0.031	0.003	1.20e-23
Height	rs16859517	T	C	0.073	0.009	3.20e-17
Height	rs16895130	G	A	0.025	0.003	2.00e-14
Height	rs16939034	T	C	0.036	0.006	1.90e-10
Height	rs16994718	C	T	0.025	0.004	6.90e-10
Height	rs17038954	T	C	0.040	0.006	1.10e-10
Height	rs17081935	T	C	0.030	0.004	5.00e-16
Height	rs17391694	T	C	0.040	0.005	4.00e-14

Height	rs1742829	T	A	0.038	0.006	6.70e-12
Height	rs17450430	T	A	0.034	0.003	6.20e-24
Height	rs17556750	A	C	0.044	0.003	0
Height	rs17783015	C	T	0.025	0.004	5.20e-10
Height	rs17806888	T	C	0.033	0.005	4.30e-12
Height	rs17807185	G	A	0.022	0.003	3.30e-13
Height	rs1797625	T	A	0.018	0.003	1.10e-09
Height	rs1812175	G	A	0.052	0.005	8.40e-30
Height	rs181338	T	C	0.029	0.003	5.70e-24
Height	rs1815314	G	A	0.035	0.003	5.50e-23
Height	rs1884897	A	G	0.038	0.003	4.70e-33
Height	rs1923367	G	C	0.029	0.003	3.20e-22
Height	rs1950500	T	C	0.031	0.003	2.70e-22
Height	rs1996422	G	A	0.022	0.003	1.30e-11
Height	rs2013265	C	T	0.027	0.003	9.20e-17
Height	rs2028067	C	T	0.033	0.004	2.00e-17
Height	rs2057291	A	G	0.020	0.003	4.90e-10
Height	rs2058092	T	C	0.017	0.003	8.40e-09
Height	rs2072268	G	A	0.021	0.003	1.70e-11
Height	rs2079795	T	C	0.045	0.003	0
Height	rs2093210	C	T	0.039	0.003	7.50e-36
Height	rs2099745	G	A	0.036	0.006	1.50e-09
Height	rs2149163	C	G	0.020	0.003	2.90e-11
Height	rs2166898	G	A	0.027	0.004	8.70e-11
Height	rs217181	T	C	0.024	0.004	2.20e-10
Height	rs2188177	T	C	0.018	0.003	8.00e-09
Height	rs2219320	T	C	0.022	0.004	3.00e-10
Height	rs2237886	T	C	0.042	0.005	1.60e-17
Height	rs227724	T	A	0.028	0.003	3.70e-19
Height	rs2284746	G	C	0.040	0.003	0
Height	rs2289195	A	G	0.042	0.003	3.00e-34
Height	rs2298265	C	T	0.030	0.005	6.90e-11
Height	rs2302580	C	T	0.029	0.004	1.20e-15
Height	rs2306694	G	A	0.047	0.006	1.20e-16
Height	rs2326458	C	A	0.022	0.004	4.50e-10
Height	rs2337143	A	G	0.018	0.003	3.20e-08
Height	rs2421992	T	C	0.031	0.004	9.40e-19
Height	rs2509133	C	T	0.020	0.003	5.30e-11
Height	rs2510396	C	G	0.029	0.004	2.60e-12
Height	rs2581830	T	C	0.025	0.003	7.60e-16
Height	rs2597513	C	T	0.042	0.005	1.10e-18
Height	rs2631676	G	A	0.028	0.004	1.50e-12
Height	rs2633761	A	G	0.017	0.003	3.70e-09
Height	rs2662027	G	T	0.032	0.005	1.40e-11
Height	rs26868	A	T	0.025	0.003	2.70e-13
Height	rs273945	C	A	0.018	0.003	2.90e-09

Height	rs2763273	C	T	0.022	0.003	1.80e-10
Height	rs2806561	A	G	0.027	0.003	2.70e-21
Height	rs2811594	G	A	0.023	0.003	3.10e-13
Height	rs2815379	G	A	0.018	0.003	2.50e-08
Height	rs2834442	A	T	0.024	0.003	5.70e-15
Height	rs2856321	G	A	0.031	0.003	1.00e-25
Height	rs2857693	G	T	0.025	0.003	5.00e-15
Height	rs2871865	C	G	0.059	0.005	8.10e-32
Height	rs2888893	C	T	0.017	0.003	7.30e-09
Height	rs291979	A	G	0.030	0.004	5.50e-18
Height	rs2974438	G	A	0.038	0.004	3.90e-26
Height	rs310421	T	G	0.028	0.003	1.40e-21
Height	rs3118905	G	A	0.044	0.004	1.60e-33
Height	rs314263	C	T	0.043	0.003	0
Height	rs316618	T	A	0.026	0.004	9.80e-13
Height	rs34651	C	T	0.042	0.006	4.20e-13
Height	rs354196	G	A	0.021	0.003	1.90e-12
Height	rs3739707	C	A	0.024	0.004	1.60e-11
Height	rs3760318	G	A	0.054	0.003	0
Height	rs3763631	C	G	0.021	0.003	3.40e-11
Height	rs3790086	C	G	0.023	0.003	9.80e-16
Height	rs3791679	A	G	0.084	0.004	0
Height	rs3802758	A	G	0.041	0.007	5.10e-10
Height	rs3807931	A	G	0.027	0.003	4.00e-21
Height	rs3812040	T	C	0.024	0.003	3.50e-13
Height	rs3814333	T	C	0.049	0.003	0
Height	rs3825199	G	A	0.054	0.004	0
Height	rs3885668	C	T	0.022	0.003	6.90e-14
Height	rs389663	T	C	0.023	0.003	1.30e-13
Height	rs3958122	T	C	0.027	0.003	1.20e-17
Height	rs4072910	G	C	0.031	0.004	9.90e-18
Height	rs42039	T	C	0.051	0.004	0
Height	rs422421	C	T	0.034	0.004	1.70e-20
Height	rs4239020	C	T	0.021	0.003	1.50e-11
Height	rs4246079	G	A	0.037	0.005	2.40e-13
Height	rs4246302	G	A	0.027	0.003	1.40e-16
Height	rs425277	T	C	0.028	0.003	4.80e-17
Height	rs4273857	A	G	0.028	0.003	6.10e-16
Height	rs429433	A	G	0.046	0.007	6.70e-11
Height	rs4320932	T	C	0.028	0.004	1.30e-11
Height	rs4325879	C	T	0.022	0.004	3.70e-10
Height	rs4344931	C	A	0.019	0.003	2.10e-09
Height	rs4369779	C	T	0.056	0.004	0
Height	rs4620037	A	C	0.035	0.004	9.90e-21
Height	rs4624820	A	G	0.018	0.003	1.80e-10
Height	rs4686904	C	T	0.022	0.003	1.00e-12

Height	rs4725061	G	A	0.020	0.003	1.50e-10
Height	rs4733724	A	G	0.050	0.004	0
Height	rs4735677	T	A	0.036	0.003	1.20e-29
Height	rs4767473	A	G	0.025	0.004	1.90e-08
Height	rs4803468	A	G	0.029	0.003	1.20e-20
Height	rs4875421	T	A	0.019	0.003	1.10e-10
Height	rs4883972	C	G	0.019	0.003	1.70e-10
Height	rs4896582	G	A	0.051	0.003	0
Height	rs4953951	C	T	0.035	0.005	4.30e-11
Height	rs4973429	G	T	0.020	0.003	1.30e-08
Height	rs4986172	C	T	0.038	0.003	1.60e-31
Height	rs540652	T	C	0.021	0.003	6.20e-13
Height	rs552707	T	C	0.047	0.003	0
Height	rs564914	T	A	0.025	0.003	4.10e-17
Height	rs5742915	C	T	0.038	0.003	1.20e-34
Height	rs5757318	T	A	0.028	0.005	3.40e-09
Height	rs584828	C	T	0.028	0.003	3.30e-20
Height	rs6020202	G	A	0.021	0.004	6.30e-09
Height	rs6061231	C	A	0.020	0.003	1.70e-10
Height	rs606452	A	C	0.043	0.004	6.40e-23
Height	rs6085662	C	G	0.021	0.003	5.20e-11
Height	rs6137287	T	C	0.020	0.003	4.80e-10
Height	rs632124	A	T	0.022	0.003	2.20e-14
Height	rs6435143	A	C	0.019	0.003	2.40e-10
Height	rs6439168	G	A	0.038	0.004	5.20e-26
Height	rs6441170	C	T	0.022	0.003	8.60e-14
Height	rs648831	T	C	0.027	0.003	3.90e-18
Height	rs6584575	A	G	0.032	0.005	1.20e-09
Height	rs6600365	C	T	0.027	0.003	9.90e-21
Height	rs6658763	C	T	0.034	0.005	5.10e-10
Height	rs6691924	T	C	0.031	0.005	4.70e-10
Height	rs6714546	G	A	0.035	0.003	2.40e-24
Height	rs6733349	T	C	0.023	0.003	7.10e-14
Height	rs6813055	A	T	0.017	0.003	5.50e-09
Height	rs6838153	G	A	0.021	0.003	7.90e-12
Height	rs6887276	G	C	0.018	0.003	3.30e-09
Height	rs6894139	T	G	0.031	0.003	4.60e-25
Height	rs6902771	T	C	0.029	0.003	1.60e-21
Height	rs6920372	G	A	0.026	0.003	5.70e-19
Height	rs6952113	G	A	0.018	0.003	1.10e-09
Height	rs6955948	T	C	0.031	0.003	8.80e-20
Height	rs6962887	T	G	0.022	0.003	9.60e-11
Height	rs6974574	T	A	0.031	0.003	2.30e-19
Height	rs7007200	G	C	0.017	0.003	4.70e-08
Height	rs7033487	T	C	0.041	0.004	3.50e-29
Height	rs7033940	G	C	0.024	0.004	3.80e-08

Height	rs7069985	G	A	0.023	0.003	1.30e-11
Height	rs7097701	C	T	0.016	0.003	2.40e-08
Height	rs7126398	C	G	0.047	0.006	1.70e-15
Height	rs7154721	T	C	0.027	0.003	1.30e-20
Height	rs7162542	G	C	0.030	0.004	7.70e-16
Height	rs7162825	T	C	0.016	0.003	2.80e-08
Height	rs7177711	A	G	0.021	0.003	1.60e-13
Height	rs720390	A	G	0.068	0.004	0
Height	rs724016	G	A	0.078	0.003	0
Height	rs7253628	G	A	0.024	0.004	6.20e-10
Height	rs7273787	G	A	0.022	0.003	3.00e-12
Height	rs731874	A	G	0.020	0.003	9.20e-10
Height	rs7319045	A	G	0.024	0.003	3.70e-15
Height	rs738288	G	A	0.019	0.003	1.50e-10
Height	rs7466269	A	G	0.033	0.003	1.70e-26
Height	rs749052	T	C	0.053	0.007	3.50e-15
Height	rs7517682	G	A	0.022	0.003	9.20e-14
Height	rs7534365	C	T	0.045	0.005	3.50e-20
Height	rs7544462	A	C	0.032	0.005	1.10e-09
Height	rs7551732	A	T	0.027	0.003	2.50e-19
Height	rs7567288	C	T	0.028	0.004	3.80e-13
Height	rs7567851	C	G	0.039	0.006	2.20e-12
Height	rs7568069	G	A	0.021	0.003	1.40e-13
Height	rs7633464	A	G	0.016	0.003	1.90e-08
Height	rs7652177	G	C	0.037	0.003	1.00e-36
Height	rs7692995	T	C	0.101	0.005	0
Height	rs7701414	G	A	0.041	0.003	0
Height	rs7716219	T	C	0.029	0.003	2.50e-21
Height	rs7823327	T	G	0.019	0.003	7.00e-11
Height	rs7849585	T	G	0.036	0.003	9.80e-29
Height	rs7870753	G	A	0.045	0.004	1.70e-37
Height	rs7899004	T	C	0.024	0.003	4.30e-17
Height	rs7971536	T	A	0.028	0.003	5.00e-18
Height	rs7980687	A	G	0.036	0.004	1.30e-21
Height	rs798497	A	G	0.057	0.003	0
Height	rs7985356	T	A	0.023	0.003	2.50e-11
Height	rs8006657	G	A	0.024	0.003	3.70e-15
Height	rs8017130	G	A	0.023	0.003	6.50e-12
Height	rs8052560	A	C	0.037	0.004	8.40e-17
Height	rs8058684	A	G	0.021	0.003	6.40e-11
Height	rs8067165	G	C	0.022	0.003	3.40e-11
Height	rs806794	A	G	0.055	0.003	0
Height	rs8097893	A	G	0.044	0.007	1.30e-10
Height	rs8103992	A	C	0.024	0.004	3.60e-10
Height	rs862034	G	A	0.030	0.003	2.60e-23
Height	rs888403	G	A	0.019	0.003	1.00e-08

Height	rs897080	C	T	0.033	0.003	2.60e-21
Height	rs9217	C	T	0.030	0.003	4.40e-23
Height	rs9291926	T	G	0.019	0.003	9.30e-10
Height	rs9292468	T	C	0.053	0.004	0
Height	rs9392918	C	T	0.041	0.003	0
Height	rs9428104	G	A	0.044	0.003	1.10e-37
Height	rs9456307	T	A	0.050	0.007	1.30e-13
Height	rs955748	G	A	0.028	0.003	4.80e-16
Height	rs958225	A	T	0.047	0.008	2.80e-09
Height	rs960006	C	T	0.021	0.003	3.00e-10
Height	rs9650315	G	T	0.057	0.005	2.50e-34
Height	rs9816693	C	G	0.031	0.004	3.60e-15
Height	rs9835332	G	C	0.028	0.003	3.00e-22
Height	rs989393	T	C	0.023	0.003	5.80e-13
Height	rs991946	C	T	0.022	0.003	6.80e-14
Height	rs991967	C	A	0.038	0.003	4.10e-32
Height	rs9929889	C	T	0.018	0.003	4.30e-08
Height	rs994533	G	C	0.031	0.003	1.90e-22
Height	rs9967417	G	C	0.037	0.003	1.20e-32
Height	rs9977276	G	T	0.023	0.004	1.30e-10
Height	rs9993613	T	G	0.030	0.003	7.80e-25
