# Supplementary material for: Body size and composition and risk of site-specific cancers in the UK Biobank and large international consortia: A mendelian randomisation study
Source: PLoS Med. 2021 Jul 29;18(7):e1003706. doi: 10.1371/journal.pmed.1003706 (PMC8320991; doi:10.1371/journal.pmed.1003706)
Supplement: S5 Table — BMI, body mass index. (PDF) [file pmed.1003706.s009.pdf]

Table S5. Supplementary analyses of the association between genetically predicted body mass index (per 1 kg/m2 increase) and cancer

		Weighted median		MR-Egger causal estimate		MR-Egger intercept	
**Cancer**	**Cases**	**OR (95% CI)**	***p*-value**	**OR (95% CI)**	***p*-value**	**Intercept**	***p*- value**
Any cancer	59 647	1.00 (0.99-1.02)	0.825	0.97 (0.94-0.99)	0.020	0.004 (0.002 to 0.007)	0.001
Any digestive system cancer	11 061	1.02 (0.99-1.06)	0.235	1.02 (0.97-1.07)	0.519	0.002 (-0.002 to 0.006)	0.388
Any non-digestive system cancer	48 586	0.99 (0.97-1.01)	0.135	0.96 (0.93-0.99)	0.004	0.005 (0.002 to 0.007)	<0.001
Breast cancer	15 695	0.99 (0.96-1.02)	0.436	0.90 (0.85-0.94)	<0.001	0.009 (0.005 to 0.014)	<0.001
Uterine cancer	2281	1.12 (1.04-1.21)	0.005	1.17 (1.04-1.31)	0.007	0.010 (0.000 to 0.021)	0.268
Cervical cancer	1973	1.01 (0.94-1.09)	0.777	0.93 (0.82-1.05)	0.224	0.001 (-0.007 to 0.010)	0.040
Ovarian cancer	1839	1.05 (0.95-1.15)	0.330	1.02 (0.90-1.16)	0.775	0.002 (-0.008 to 0.013)	0.698
Prostate cancer	10 506	0.99 (0.96-1.03)	0.795	0.98 (0.92-1.04)	0.446	0.002 (-0.008 to 0.013)	0.753
Testicular cancer	747	0.94 (0.83-1.07)	0.377	0.88 (0.73-1.07)	0.207	0.010 (-0.007 to 0.026)	0.243
Bladder cancer	3326	1.00 (0.93-1.07)	0.935	0.92 (0.84-1.01)	0.078	0.011 (0.003 to 0.019)	0.010
Kidney cancer	1741	1.01 (0.94-1.10)	0.727	0.94 (0.83-1.07)	0.348	0.010 (-0.001 to 0.021)	0.060
Brain cancer	1057	1.01 (0.89-1.15)	0.853	0.95 (0.80-1.13)	0.229	0.006 (-0.008 to 0.021)	0.401
Head and neck cancer	1983	0.94 (0.86-1.01)	0.103	0.85 (0.76-0.96)	0.006	0.013 (0.003 to 0.023)	0.010
Thyroid cancer	384	1.08 (0.89-1.30)	0.435	1.05 (0.81-1.37)	0.705	-0.002 (-0.024 to 0.021)	0.797
Oesophageal cancer	1228	1.12 (1.00-1.26)	0.042	1.20 (1.03-1.40)	0.022	-0.009 (-0.021 to 0.005)	0.229
Stomach cancer	994	1.11 (0.98-1.25)	0.096	1.14 (0.97-1.34)	0.117	-0.001 (-0.015 to 0.013)	0.923
Colorectal cancer	6995	1.02 (0.97-1.07)	0.494	1.02 (0.95-1.09)	0.558	-0.001 (-0.007 to 0.005)	0.776
Pancretic cancer	1747	1.05 (0.97-1.15)	0.237	0.98 (0.86-1.10)	0.702	0.008 (-0.003 to 0.018)	0.137
Liver cancer	463	1.09 (0.93-1.28)	0.310	0.97 (0.77-1.23)	0.821	0.014 (-0.006 to 0.034)	0.932
Biliary tract cancer	604	0.98 (0.85-1.14)	0.807	0.99 (0.80-1.22)	0.936	0.007 (-0.011 to 0.025)	0.676
Melanoma	5691	0.96 (0.92-1.01)	0.162	1.00 (0.93-1.07)	0.889	-0.001 (-0.007 to 0.005)	0.739
Lung cancer	4231	1.09 (1.03-1.15)	0.003	0.99 (0.91-1.08)	0.748	0.009 (0.001 to 0.016)	0.121
Leukaemia	1825	1.02 (0.93-1.11)	0.725	0.93 (0.82-1.04)	0.206	0.010 (-0.000 to 0.020)	0.057
Non-Hodgkin lymphoma	2878	1.03 (0.96-1.10)	0.454	1.05 (0.95-1.15)	0.354	-0.001 (-0.009 to 0.007)	0.842
Multiple myeloma	930	1.07 (0.95-1.21)	0.266	1.06 (0.90-1.26)	0.467	-0.006 (-0.020 to 0.008)	0.426
