# Supplementary material for: Body size and composition and risk of site-specific cancers in the UK Biobank and large international consortia: A mendelian randomisation study
Source: PLoS Med. 2021 Jul 29;18(7):e1003706. doi: 10.1371/journal.pmed.1003706 (PMC8320991; doi:10.1371/journal.pmed.1003706)
Supplement: S6 Table — (PDF) [file pmed.1003706.s010.pdf]

Table S6. Supplementary analyses of the association between genetically predicted height (per 1 standard deviation increase) and cancer

		Weighted median		MR-Egger causal estimate		MR-Egger intercept	
**Cancer**	**Cases**	**OR (95% CI)**	***p*-value**	**OR (95% CI)**	***p*-value**	**Intercept**	***p* value**
Any cancer	59 647	1.10 (1.05-1.15)	<0.001	1.11 (1.02-1.21)	0.019	-0.001 (-0.004 to 0.002)	0.579
Any digestive system cancer	11 061	1.11 (1.01-1.22)	0.023	1.11 (0.94-1.31)	0.225	-0.000 (-0.006 to 0.005)	0.930
Any non-digestive system cancer	48 586	1.07 (1.02-1.12)	0.011	1.11 (1.01-1.22)	0.031	-0.001 (-0.004 to 0.002)	0.544
Breast cancer	15 695	1.03 (0.95-1.11)	0.545	1.17 (0.99-1.37)	0.062	-0.001 (-0.006 to 0.004)	0.603
Uterine cancer	2281	1.11 (0.91-1.37)	0.304	1.20 (0.83-1.72)	0.340	-0.003 (-0.014 to 0.008)	0.608
Cervical cancer	1973	0.93 (0.75-1.16)	0.515	1.00 (0.67-1.50)	0.992	-0.003 (-0.014 to 0.008)	0.608
Ovarian cancer	1839	1.17 (0.94-1.45)	0.169	1.25 (0.86-1.82)	0.247	-0.001 (-0.013 to 0.010)	0.867
Prostate cancer	10 506	1.07 (0.96-1.18)	0.220	1.21 (0.99-1.49)	0.066	-0.006 (-0.013 to 0.001)	0.055
Testicular cancer	747	1.04 (0.74-1.46)	0.830	0.51 (0.26-1.02)	0.056	0.022 (0.001 to 0.044)	0.040
Bladder cancer	3326	1.03 (0.74-1.46)	0.830	0.51 (0.26-1.02)	0.056	0.002 (0.001 to 0.044)	0.314
Kidney cancer	1741	1.00 (0.79-1.25)	0.986	0.96 (0.64-1.45)	0.862	0.007 (-0.006 to 0.020)	0.276
Brain cancer	1057	1.15 (0.86-1.54)	0.341	1.56 (0.94-2.58)	0.084	-0.012 (-0.028 to 0.003)	0.123
Head and neck cancer	1983	0.97 (0.79-1.19)	0..764	0.83 (0.59-1.18)	0.309	0.009 (-0.002 to 0.020)	0.123
Thyroid cancer	384	1.49 (0.93-2.38)	0.098	1.89 (0.85-4.19)	0.120	-0.014 (-0.039 to 0.011)	0.283
Oesophageal cancer	1228	1.15 (0.88-1.51)	0.311	1.00 (0.62-1.59)	0.985	0.001 (-0.014 to 0.016)	0.899
Stomach cancer	994	1.03 (0.77-1.38)	0.846	0.93 (0.57-1.53)	0.782	0.001 (-0.015 to 0.017)	0.893
Colorectal cancer	6995	1.07 (0.95-1.20)	0.254	1.03 (0.83-1.28)	0.802	0.002 (-0.005 to 0.009)	0.553
Pancretic cancer	1747	1.13 (0.90-1.41)	0.303	1.16 (0.78-1.74)	0.458	-0.003 (-0.016 to 0.010)	0.644
Liver cancer	463	1.15 (0.75-1.77)	0.509	0.89 (0.42-1.87)	0.760	0.007 (-0.016 to 0.030)	0.567
Biliary tract cancer	604	1.47 (1.00-2.15)	0.049	1.46 (0.77-2.75)	0.249	-0.000 (-0.020 to 0.020)	0.986
Melanoma	5691	1.05 (0.93-1.20)	0.430	1.07 (0.84-1.36)	0.601	-0.001 (-0.009 to 0.006)	0.673
Lung cancer	4231	1.03 (0.89-1.19)	0.699	1.15 (0.89-1.50)	0.281	-0.002 (-0.010 to 0.006)	0.623
Leukaemia	1825	1.04 (0.84-1.29)	0.725	1.10 (0.76-1.58)	0.622	-0.001 (-0.013 to 0.010)	0.846
Non-Hodgkin lymphoma	2878	0.97 (0.81-1.15)	0.708	1.27 (0.95-1.71)	0.105	-0.005 (-0.014 to 0.004)	0.105
Multiple myeloma	930	1.02 (0.74-1.39)	0.925	0.66 (0.38-1.16)	0.147	0.016 (-0.001 to 0.034)	0.177
