# Supplementary material for: Body size and composition and risk of site-specific cancers in the UK Biobank and large international consortia: A mendelian randomisation study
Source: PLoS Med. 2021 Jul 29;18(7):e1003706. doi: 10.1371/journal.pmed.1003706 (PMC8320991; doi:10.1371/journal.pmed.1003706)
Supplement: Attachment — Submitted filename: 2020_Nov_10 BMI Cancer_PLoS_Response_SB.docx [file pmed.1003706.s011.docx]

**Requests from the editors:**

E1. Background- please provide brief context for this study, include 1-2 sentence of why this work may be important

*Thank you for the comments. As recommended, we have provided a brief context for this study: “This study is important to elucidate the causal role of body composition for site-specific cancers, and so extend and focus the evidence base for targeted public health strategies.”*

E2. Please structure the abstract to: background, methods and findings, conclusions

*This has been changed as requested.*

E3. Please provide participant demographics

*Patient demographics for UK Biobank are shown in Table 1.*

E4. Please provide additional details about the analyses carried out in the methods and findings section.

*We have added: “Similar results were observed in analyses using the weighted median and MR-Egger methods.”*

E5. In the last sentence of the Abstract Methods and Findings section, please describe the main limitation(s) of the study's methodology.

*We have added: “The main limitations are the assumption that genetic associations with cancer outcomes are mediated via the proposed risk factors, and that estimates for some lower frequency cancer types are subject to low precision.”*

E6. Please add p values along with 95% CI as needed

*These have been added to the abstract.*

E7. Please temper conclusions by adding “our results show” or similar

*We have edited the manuscript as suggested: "Our results show the evidence for BMI…* ***"***

E8. At this stage, we ask that you include a short, non-technical Author Summary of your research to make findings accessible to a wide audience that includes both scientists and non-scientists. The Author Summary should immediately follow the Abstract in your revised manuscript. This text is subject to editorial change and should be distinct from the scientific abstract. Please see our author guidelines for more information: <https://journals.plos.org/plosmedicine/s/revising-your-manuscript#loc-author-summary>

*We have included this in the manuscript.*

E9. Please ensure that the study is reported according to the STROBE guideline, and include the completed STROBE checklist as Supporting Information. Please add the following statement, or similar, to the Methods: "This study is reported as per the Strengthening the Reporting of Observational Studies in Epidemiology (STROBE) guideline (S1 Checklist)."

*This statement has been added to the end of the methods section.*

E10. The STROBE guideline can be found here: <http://www.equator-network.org/reporting-guidelines/strobe/>. When completing the checklist, please use section and paragraph numbers, rather than page numbers.

*We have completed this checklist and enclosed this in the submission.*

E11. Did your study have a prospective protocol or analysis plan? Please state this (either way) early in the Methods section.

a) If a prospective analysis plan (from your funding proposal, IRB or other ethics committee submission, study protocol, or other planning document written before analyzing the data) was used in designing the study, please include the relevant prospectively written document with your revised manuscript as a Supporting Information file to be published alongside your study, and cite it in the Methods section. A legend for this file should be included at the end of your manuscript.
b) If no such document exists, please make sure that the Methods section transparently describes when analyses were planned, and when/why any data-driven changes to analyses took place.
c) In either case, changes in the analysis-- including those made in response to peer review comments-- should be identified as such in the Methods section of the paper, with rationale.

*We have added the statement: “Our analysis did not have an explicit pre-specified analysis plan. The analysis was conducted similarly to previous published efforts for investigating the causal relationships of BMI, FMI, and FFMI with cardiovascular diseases. In response to comments from peer reviewers, we made a number of changes to the analysis: we changed the overall cancer outcome from including all cancers to including any of the 22 named site-specific cancers, we updated the genetic variants used as instrumental variables to those from the latest GWAS for the relevant risk factor, and we added analyses based on large-scale consortia for lung, ovarian, breast, uterine and prostate cancer.”*

E12. Please avoid using words such as “obese” and “overweight” throughout the text. Please amend to “with obesity” and “with overweight” throughout in line with people first principles.

*This has been changed in the text.*

E13. Please add a space between text and reference brackets throughout

*This has been amended.*

E14. Methods- please provide further details on the data sources used, including names of registries or sources of electronic medical records

*We have added the text: “Cancer outcomes were obtained from electronic health records, hospital episodes statistics (HES) data, the national cancer registry, death certification data and self-reporting validated by nurse interview.”*

E15. Table 1 please add units to the row “Age”

*The units (years) have been added.*

**Comments from the reviewers:**

**Reviewer #1:**

In the present study, the authors analyzed the effect of body size and composition on cancer risk using UK Biobank data. Even though a wide-angled MR investigation was performed to evaluate the impact of obesity on cancer outcomes, there are several deficiencies that need to be clarified by the authors, amongst which some of these issues limited the interpretability of the findings

A1.      In the methods section, it was unclear how the quality control was performed. A clearer flowchart was needed, at least, in the supplementary.

*As recommended, we have added a flowchart (Supplementary Figure S1).*

A2.      In the statistical analysis, it is not clear what kind of Mendelian randomized analysis was adopted in this study? And which R package is used for this analysis?

*We have now clarified which MR analyses were conducted and the Stata and R packages used.*

*"Associations of genetically-predicted BMI and height with the 22 site-specific cancers and overall cancer for the UKBB cohort were obtained using the random-effects inverse-variance weighted method [39]. We performed additional analyses using the weighted median [39] and MR-Egger [40] methods. The analyses of FMI and FFMI were based on the multivariable random-effects inverse-variance weighted method with both exposures included in the same model."*

*"Statistical analyses were performed in Stata/SE 14.2 using the mrrobust package and R 3.6.0 software using the MendelianRandomization package. "*

A3.      Because a recent Mendelian randomized study has shown a J shaped relation between BMI and all-cause mortality, is there a non-linear association between BMI and cancer risk? If so, will it affect the results of this study?

*Several large observational studies and pooled analyses of cohort studies have suggested a linear dose-response relationship between BMI and different cancers (e.g., PMID: 17986716, 12711737, 15734973, 23733771, and 31113819). While a recent Mendelian randomization analysis indicated a J-shaped relationship between BMI and all-cause mortality (*[*https://www.bmj.com/content/364/bmj.l1042*](https://www.bmj.com/content/364/bmj.l1042)*), the same analysis showed no strong evidence of a non-linear relationship between BMI and cancer mortality (Figure 5C of the reference) or incident cancer (Supplementary Figure 4B of the reference). Hence we do not expect non-linearity to be substantial in this case.*

*We have added to the paragraph on limitations: “Our analysis assumes a linear relationship between the risk factors and the outcome. Quantitative estimates may be misleading if the true relationship is non-linear; although estimates are still reflective of the presence and direction of the population-averaged causal effect.”*

A4.      "The correlation of BMI with FMI was 0.84, and with FFMI was 0.66. The correlation between FMI and FFMI was 0.14." in this study, whether the instrumental variable of BMI is independent of FMI? Does the relation among those variables affect the findings of this study?

*High correlation between risk factors means it is difficult to differentiate between the risk factors in a multivariable analysis. As the correlation between the genetic associations with FMI and FFMI is relatively low, estimates from the multivariable analysis for FMI and FFMI are likely to be reliable. We have added a sentence to the manuscript to clarify this point.*

A5.      Could the authors explain why there was a so strong effect of fat mass index on the stomach risk (OR=4.23) and liver cancer (OR=4.28)?

*These strong estimates in the previous submission were relatively imprecise. With the new instruments for fat mass and fat-free mass indices (577 SNPs as compared with 81 SNPs in previous analyses) and updated information on cancer (more cancer cases), the effect estimates for the associations of genetically-predicted fat mass index with stomach and liver cancer are smaller.*

A6.      The incident events for most cancers were too small to perform a genetic analysis, as a result, the associations was not robust as far as I can see.

*We agree there is low precision estimates for certain site-specific cancers. We have highlighted this in the limitations section of the discussion: “… estimates for some lower frequency cancer types are subject to low precision.”. However, the strongest association for genetically-predicted BMI is with liver cancer, which only has 463 cases. This illustrates that power to detect a causal effect can be sufficient even for a relatively uncommon cancer.*

A7.      How about the direct and indirect effects of genetic components, BMI/ FMI/ FFMI, and cancer risk?

*The assumption in Mendelian randomization is that genetic variants satisfy the assumptions of an instrumental variable. In particularly, this means that the genetic association with the outcome is mediated solely via the proposed risk factor (or risk factors). Therefore by assumption, there is no direct effect of the genetic variants on the outcome. This assumption is now explicitly stated in the abstract and the limitations paragraph of the discussion: “The main limitation is the assumption that the genetic associations with cancer risk are mediated via the proposed risk factors.”*

A8.      The number of overall cancer seems inconsistent with cancer-specific events according to the figures.

*Previously, our overall cancer analysis included all cancer outcomes. However, following a comment from a reviewer (B4 from Reviewer 2 below), we now only include the 22 named site specific cancers in our definition of overall cancer.*

**Reviewer #2:**

*ºWhat are the main claims of the paper and how significant are they for the discipline?*
This study applied mendelian randomisation to explore the causal impact of BMI and height on all cancer, and site specific cancers. In addition to BMI the study also considers the impact of facets of BMI - fat and fat-free mass index.

The main finding is that higher BMI has a causal but not consistent role in some site specific cancers e.g. increasing the risk of some, and protective for others. There was no evidence for a causal risk for overall cancer risk, contrary to previous reports (see following). In line with previous reports they found that genetically predicted increases in height is causally associated with increased rates of all cancers, and the majority of site specific cancers.

*Significance for the discipline:* This heterogeneity of direction with respects to BMI and site specific cancers is interesting but caution should be made in suggesting that as a result nuance should exist in regards to the obesity as a risk factor for specific cancers (discussion, third to last paragraph). Setting aside that a similar study using UK Biobank data (see following) found there was a consistent increase in risk for all cancer and all-cancer mortality for a genetically predicted increase in BMI (and thus reducing BMI would be expected to reduce the overall cancer burden in the population even if some subsets might rise), if the true causal estimate is that higher BMI does not increase overall risk for cancer as reported here, within the site specific cancers the protective effect from higher BMI would have to be balanced against both the increase in other cancers, and other BMI related morbidities. That is, the likely health advice would be to reduce BMI even in populations at risk of cancers where BMI may be protective. This is especially true given two of the cancers with reported reduced odds with higher BMI (melanoma and prostate) are either relatively less likely to kill compared to those that increase in risk with higher BMI, and/or have a range of interventions that could counter the potential increased risk (e.g. increased use of sunscreen for melanoma). That is, the advice would likely still be to reduce BMI in concert with appropriate cancer specific risk reduction strategies. Even for cancers with more severe prognosis, or fewer protective strategies (e.g. breast) the likely answer would be increased screening in those with genetically predicted higher BMI and still reduce BMI.
B1. Action - The discussion should better explain what role nuance would play in BMI messaging in light of this paper

*Thank you for this very interesting point. We have explained this in the text "Previous public health recommendations have advocated obesity as a generic risk factor for cancer prevention. While our research supports a causal role of obesity in driving and protecting against certain cancers, it suggests differential effects of BMI and obesity in different malignancies which should be explored further. A more nuanced message public health message with regards to obesity as a risk factor for digestive system cancers may be more appropriate.”*

*ºAre the claims properly placed in the context of the previous literature? Have the authors treated the literature fairly?*

There are some important omissions in the reported literature that impact both the perceived novelty of this work, and its interpretation.

Gharahkani 2019 PMID: 30733581 performs a very similar analysis in the same UK Biobank dataset, using a larger BMI instrument to test both site specific and overall cancer risk. There are differences in the new analysis, but these should be interpreted with respect to that publication. This is especially relevant as this study found obesity is associated with all cancer, unlike this analysis, and this point has relevance with respects to the papers discussion.

*Many thanks for this. We have included this recent MR analyses based on UKBB in our discussion and compared their findings with our study.*

While not significant at P< 0.05 Levy 2017 PMID: 28804972 reported an OR of 0.848 (0.658-1.093) for testicular cancer given a 1sd increase in BMI; while this is smaller than the causal estimate in this paper especially given the degree of change in BMI the Cis are wide for this estimate and likely overlap with the estimate and this should be discussed in the paper with respects to novelty

*After increasing the number of SNPs used as instrumental variables for BMI, we found this negative association between elevated BMI and testicular cancer persisted but did not cross the null.*

The discussion with respect to a positive association between height and overall cancer risk, and site specific cancer, while citing a similar paper (Ong et al 2018, ref 57) for specific cancers, does not note that ref 57 performed a very similar analysis in the same UK Biobank dataset and found consistent results; this should be addressed/contrasted in terms of novelty and what this study adds/improves.

*We report similar findings from this MR, though also include a larger cohort from the breast cancer consortia which strengthens the findings of a positive association between height and breast cancer risk.*

The observation for BMI on melanoma is at odds with the uncited Dusingize 2020 PMID: 32068838 and this difference should be addressed; Dusingize 2020 also investigated height and found a consistent result for melanoma as this study.

*Our updated results have shown no association between BMI and melanoma, but negative association between FMI and melanoma and inverse relationship with FFMI. This is suggestive that body composition and adiposity may play a specific role in melanoma carcinogenesis.*

B2 Action: Cite these papers, and check for any additional relevant papers not reported here (I may have missed others) and then address them in their publication with respects to novelty, consistency and differences/advances.

*Many thanks for these suggestions - we have incorporated these papers into the Discussion.*

*ºDo the data and analyses fully support the claims? If not, what other evidence is required?*

Partially:

More powerful BMI and height instruments are available using UK Biobank data or more recent GWAS meta-analyses e.g. Yengo 2018 PMID: 30124842 reports potential instruments explaining 6% of BMI and 24% of height variance. Gharahkani 2019 PMID: 30733581 constructed two instruments for BMI using cancer free UK Biobank participants that explained 4 or 7% of BMI variance. This would increase power for site specific estimates compared to those generated using their current instrument, which explains 1.6% of trait variance. While their power for height is greater (and arguably already sufficient), likewise Yengo 2018 or UK Biobank provides even more powerful instruments for height.

B3 Action: The authors should take advantage of data published or in hand to construct more powerful BMI/height instruments to improve precision/power.

*We have updated the BMI analysis to incorporate 312 SNPs from Pulit et al 2019 and use of consortia cancer data for lung, breast, ovarian, endometrial, and prostate cancer to improve precision.*

The overall cancer measure appears to include non-melanoma cancers (keratinocyte cancers, basal and squamous cell carcinoma) as the overall cancer N is ~20k more than individual site count, and there is ~ this many ICD C44 cancer cases in UK Biobank. However table S1 does not report if C44 is included in the overall cancer phenotype. Observational studies suggest an inverse relationship between BMI and non-melanoma skin cancer (e.g. Zhou 2016 PMID: 27898109); if this is a causal effect, given how common non-melanoma skin cancers are in UK Biobank, this may explain why in this study BMI was associated with no increase in all-cancer while Gharahkani 2019 PMID: 30733581, which excluded these cancers, did. That is, the overall cancer estimate here may (crudely) be the sum of a protective effect on non-melanoma skin cancer, and in aggregate an increase for all other cancers.

This is an important distinction as non-melanoma skin cancers are very rarely lethal, are in general easily treated, and adequately controlled by sun smart campaigns - that is, even if BMI is (causally) protective for non-melanoma skin cancer such that the total number of cases of cancer does not change as BMI increases, trading off higher rates of NMSC for all other cancers is likely to be an acceptable decision.

B4 Action: the impact of including, or excluding, non-melanoma skin cancers in all cancer estimates should be reported.

*Our analysis of overall cancer this now only includes the combined 22 site cancers - which does not include non-melanoma skin cancer. We have made this change as we agree with the reviewer that these are the most clinically relevant cancers.*

B5 Action/comment: For the discussion hypothesis that increased IGF1 expression may underlie the causal effect of height - is this amendable to analysis with summary statistic data-based Mendelian randomization (SMR)? GTex reports eQTLs for this gene

*We have added a summary of our recently published MR investigation looking at IGF-1 and cancer risk to the Discussion. This found a positive association of genetically-predicted IGF-1 with colorectal cancer, but no association of genetically-predicted IGF-1 with overall cancer, suggesting that IGF-1 does not substantially mediate the effect of height on cancer risk.*

*ºPLOS Medicine encourages authors to publish detailed methods as supporting information online. Do any particular methods used in the manuscript warrant such publication? If a protocol is already provided, for example for a randomized controlled trial, are there any important deviations from it? If so, have the authors explained adequately why the deviations occurred?*

Methods are sufficient barring other comments.

*°Is this paper outstanding in its discipline? If yes, what makes it outstanding? If not, why not?*

The paper is interesting but given the overlap with previous work is not in its current form outstanding.

*ºDoes the study conform to any relevant guidelines such as CONSORT, MIAME, QUORUM, STROBE, and the Fort Lauderdale agreement?*

N/A

*ºAre details of the methodology sufficient to allow the experiments to be reproduced?*

Action: related to above the methods should clarify how the overall cancer phenotype was constructed (e.g. does it include additonal cancers/ICD codes to those reported in sup table 1

*ºIs any software created by the authors freely available?*

N/A

*ºIs the manuscript well organized and written clearly enough to be accessible to non-specialists?*

In general the paper is well written and clear; two minor points:

B6 1)       Results paragraph 1 final sentences - are the reported correlations phenotypic, genetic etc?

*We have clarified this in the text.*

B7 2)      Results sub section BMI and cancer risk - the CIs for prostate cancer in line 4 don't match the figure/P-value.

*Many thanks for highlighting this - it has been corrected.*

**Reviewer #3:**

The MR analyses run in the paper are pretty standard. I have a few comments on the Mendelian Randomization analyses that the authors have performed:

C1. One concern I have is that GWAS data for all the traits involved (the three risk factors and various cancer traits) are using the same UK Biobank data. A consequence is that for the exposure and outcome, some individuals are shared thus the summary statistics are also correlated due to non-heritable factors. In other words, for any specific SNP, the estimation of its effect on the exposure and outcome are not independent, which may bring bias to the MR analysis.

*We take the reviewer’s point. There is a compromise to be made between selecting variants based on the largest available GWAS investigation, and selecting variants based on a GWAS investigation that excludes UK Biobank. Increasingly, these options are mutually exclusive. Following comments from reviewers, we have chosen to select variants based on the largest available GWAS investigation. This does risk bias due to sample overlap and winner’s curse. However, results were replicated in all analyses based on consortium data, suggesting that bias is not substantial.*

*We now discuss this point in the limitations paragraph of the Discussion: “Selection of genetic variants was based on datasets that include UK Biobank participants, and for FMI and FFMI, on a dataset comprised solely of UK Biobank participants. This may lead to bias due to sample overlap and winner’s curse. However, results were similar when genetic associations with cancers were taken from independent consortia.*”.

C2. Related the question 1, I think to make the authors' conclusion more convincing, they can check whether than see replicable effects of BMI/FMI/height on cancers using BMI/FMI and cancer GWAS data from other cohorts (either European ancestry or other ethics).

*We have performed MR analysis on consortia data for lung, breast, ovarian, endometrial, and prostate cancer. Results are seen to replicate for all outcomes.*

C3. In the discussions, the authors explained why BMI/height can have a causal effect on certain cancers. For height, the authors explained that its effect on height can be mediated by IGF1. I'm wondering if there are any GWAS data available for IGF1 (or similar traits) so that MR analyses can be performed to support this explanation. In general, I think the authors may need to provide more evidence to support their conclusions instead of using only the UK Biobank data and MR.

*We have added a summary of our recently published MR investigation looking at IGF-1 and cancer risk to the Discussion. This found a positive association of genetically-predicted IGF-1 with colorectal cancer, but no association of genetically-predicted IGF-1 with overall cancer, suggesting that IGF-1 does not substantially mediate the effect of height on cancer risk.*

C4. Since the authors look across about 20 cancers, I think multiple testing adjustment are needed. Since the significant p-values are not that small, I'm wondering if there are still many interesting signals left after adjustment.

*As the number of cases and thus statistical power differed between analyses, we did not set a fixed threshold for statistical significance. We prefer to state the results clearly and let the reader judge the level of statistical evidence, rather than to provide a binary categorization of results as “significant” or “not significant”. We note that several of the p-values are less than 0.001, and so would still be considered “significant” after correction for multiple testing.*

C5. I recommend also performing MR reversing the role of cancer and BMI/height to see whether there is reserve causation (especially for BMI/FMI/FFMI).

*While the reviewer’s suggestion is interesting, this paper is already long, and so we are reluctant to add substantial additional content. Additionally, estimation of the effect of cancer on BMI by MR would require genetic variants which have primary and specific effects on cancer. It would be difficult to argue that any association of genetic predictors of cancer with BMI is necessarily mediated via cancer. Hence, MR analyses in the reverse causal direction would be unreliable.*

C6.  In the section of "statistical analysis", the authors stated that "We performed sensitivity analyses using weighted median and MR-Egger", however I don't think weighted median and MR-Egger are sensitivity analyses, these are just two other MR methods.

*We have removed the phrasing sensitivity analysis.*

**Reviewer #4:**

D0. It was a pleasure to comment on this well-conducted and concisely written study. My main concern is limited reference to the most recent literature on adiposity and cancer; in particular, meta-analyses/umbrella reviews of observational studies.

Background

D1. Because the focus is on obesity-related traits and cancer, I don't see the necessity to state that obesity is a risk factor for cardiovascular, liver, and musculoskeletal diseases.

*We have removed this sentence as suggested.*

D2. I suggest shortening the citation of existing MR studies on body fatness and cancer and instead highlight the available evidence from observational studies. Based on grading by the World Cancer Research Fund and American Cancer Institute, strong evidence from meta-analyses of observational studies exists for the BMI and waist circumference and increased risk of postmenopausal, colorectum, endometrium, ovary, kidney, liver, gall bladder, stomach, esophagus, and pancreas, and moderate evidence exists for an association with cancers of the mouth, pharynx, larynx, prostate (advanced), male breast, and diffuse large B-cell lymphoma. Consider citing the most current grading of observational evidence from WCRFI/AICR/IARC [1-3] and umbrella reviews (e.g., [4-6]).

*Many thanks for the suggestion - we have stated more site-specific cancers associated with raised BMI. "In particular, strong positive associations have been observed between BMI and risk of colorectal, stomach, oesophagus, liver, gallbladder, breast, endometrium, ovarian, kidney and pancreatic cancer [9–11]".*

D3. Could you be more specific which biases plaque observational research on obesity and cancer? Key biases include confounding by smoking [7-9] and reverse causation by subclinical and prevalent disease (cancer, cvd)[7, 10-12].

*Many thanks - we have added and referenced these studies in the introduction "However, traditional epidemiological studies are subject to confounding factors, such as smoking, and reverse causation due to subclinical disease. Thus, the true relationship between obesity and cancer remains unclear."*

Methods

D4. Since adjustments (e.g. for waist in a GWAS of BMI) can bias the MR estimate, please report whether exposure GWAS adjusted for covariables [13-15].

*These associations were only adjusted for age, sex, and principal components, and were not adjusted for other variables. We have added to the manuscript: “Genetic associations […] were adjusted for age, sex, and genetic principal components.”.*

D5. There's a larger GWAS[16] on BMI than the GWAS by Locke - I am sure if summary data excluding UKBB is available. Reliance on BMI as a measure of general adiposity is limited by its inability to discriminate fat mass and lean body mass [12] and this can be more problematic in subclinical diseased individuals who often experience unintentional weight loss. I, therefore, welcome the use of fat mass and fat-free mass as additional exposures.

*As per the response to point B3, we have updated the choice of genetic variants used in the analysis. We agree with the reviewer that it is interesting to look at the role of FMI and FFMI.*

D6. I doubt the validity of defining cancer cases based on 'self-report validated by a nurse', especially for less common cancers and histotypes. Can the authors provide data on validity? I suggest limiting outcome definition to cancer registry, outpatient and inpatient records, and death certificates.

*Of the 59 647 cancer events, 55 674 events were defined based on cancer registry, outpatient and inpatient records, and death certificates. Thus, the vast majority of cancer events are not self-reported. We have performed sensitivity analyses with self reported events excluded. Results for site-specific cancers are similar but generally are marginally less precise, whereas the association between genetically-predicted BMI and overall cancer is slightly attenuated (Supplementary Figure S3).*

D7. I assume that using outcome data from the largest available GWAS meta-analyses would provide more cases and increase power. For example, a recent breast cancer GWAS[17] had 122,977 cases of European descent (another one[18] had 133,384 cases), compared to 13,666 cases included in the present MR study. Likewise, the largest available GWAS on lung carcinoma[19, 20] contains 10 times the number of cancer than the UK Biobank. I suggest adding replication analyses using the largest available cancer GWAS data. This would also address and circumvent possible biased introduced by weak instrumentation in 1-sample MR, winner's-curse (fmi,ffmi), and sample overlap. Although this could introduce new bias that should be discussed (e.g.,selection/survival bias). Can endometrial cancer be added as an outcome?

*We have now performed MR analysis on consortia data for lung, breast, ovarian, endometrial and prostate cancer. Note that in UK Biobank, endometrial cancer is included in uterine cancer.*

D8. The 'overall cancer' outcome lumps together obesity-related cancers with cancers that are not affected by body fatness. I suggest omitting the total cancer analyses.

*We agree that the analysis for overall cancer could possibly mask risk-increasing, risk-decreasing, and risk-neutral effects for different cancers. However, this analysis is important from a public health perspective, as the decision to target BMI as a risk factor for cancer depends on its effect on overall cancer, not its effect on one site-specific cancer. To minimize the potential attenuating effect of non-melanoma and less clinically important cancers, we have restricted the overall cancer analysis to the 22-site specific cancers.*

D9. MR-Egger is sensitive to influential points in the regression[21] and point estimates are often imprecise[22, 23], as can be witnessed in Table S4. MR Egger is therefore not generally recommended as a preferred sensitivity analysis method [23-25]. I suggest to follow Slob and Burgess and report one of the three robust MR method classes discussed in [23] and omit MR Egger estimates. Consider adding E-values [26], variant-outcome associations (fewer assumptions [27, 28]), and negative controls [29].

*While we agree with the reviewer, the MR-Egger method is commonly used in practice. It makes a different assumption to other robust methods for Mendelian randomization, and hence is a useful method to present in contrast to the IVW and weighted median methods, particularly in this context as the number of genetic variants is large. Although we could have made different choices of robust methods to present, we view the choice of methods as a question of preference rather than a question of validity. As to the reviewer’s other suggestions, variant—outcome associations would be difficult to interpret given the large number of variants in each analysis, and we do include height as a control risk factor (although a positive control rather than a negative control). Given the volume of material already in the paper, we are reluctant to increase the volume of results presented unless there is a compelling reason to do so.*

Discussion

D10. MR studies can, like observational studies, be subject to bias (pleiotropy - in particular when the biology of the variant-exposure association is ill-defined, population stratification, selection bias etc). Applying different study designs and analytical approaches may in the long run converge to provide triangulating evidence [30-32] and help strengthen answering causal relationships. I suggest separating the evidence from observational and MR studies; followed by a "grading" of the combined observational and MR evidence. It would be helpful to first summarize and grade the observational evidence based on the best available meta-analyses/umbrella reviews, possibly adapting the extensive work of WCRFI/AICR/IARC, then raise the limitations of observational studies (i.e., potential biases introduced by smoking and reverse causation by pre-existing disease). Then summarize the available MR-studies and illustrate where findings of observational and MR studies converge or diverge. Also, it would help to provide more details on the strengths and limitations of the available MR studies on obesity-traits and cancer. In particular, the number of cases or power achieved by the present MR study and previous MR studies should be discussed. Several recent large MR studies [33-37] that are not cited.

*Many thanks for the comments. We have incorporated this into our discussion, including the umbrella reviews and meta-analysis, and the limitations of traditional observational studies which may explain divergence with MR studies. We have also included the MR studies suggested in our discussion.*

D11. For breast cancer, according to observational research, being overweight or obese as an adult before menopause decreases the risk of premenopausal cancer of the female breast, but greater weight gain in adulthood increases the risk of postmenopausal breast cancer [1]. Observational research has provided the strongest evidence for obesity and postmenopausal [6]. The differential effects on pre- and postmenopausal breast cancer require further investigation A recent MR [36] study found that childhood adiposity might protect against adult breast cancer but did not consider breast cancer age of onset. Body composition is s time-varying exposure and the difficulties of MR to handle such exposures should be more clearly highlighted.

*This is a very interesting point which we have also observed. We have included the sentence: "These MR results contrast with observational study findings which have demonstrated adult obesity to be associated with increased risk of postmenopausal breast cancer.".*
